# Structural diversity in the host–guest complexes of the antifolate pemetrexed with native cyclodextrins: gas phase, solution and solid state studies

**DOI:** 10.3762/bjoc.13.222

**Published:** 2017-10-25

**Authors:** Magdalena Ceborska, Magdalena Zimnicka, Aneta Aniela Kowalska, Kajetan Dąbrowa, Barbara Repeć

**Affiliations:** 1 Institute of Physical Chemistry, Polish Academy of Sciences, Kasprzaka 44/52, 01-224 Warsaw, Poland; 2 Institute of Organic Chemistry, Polish Academy of Sciences, Kasprzaka 44/52, 01-224 Warsaw, Poland

**Keywords:** antifolate, cyclodextrin, hydrophobic interactions, inclusion complexes, pemetrexed, supramolecular chemistry

## Abstract

The complexation of the antifolate pemetrexed (PTX) with native cyclodextrins was studied. This process, along with the findings gathered for the structurally related folic acid was treated as a model for exploiting host–guest interactions of this class of guest molecules in the gas phase, in solution and in the solid state. Mass spectrometry was employed for the investigation of the architecture and relative gas-phase stabilities of these supramolecular complexes. The mode of complexation was further tracked by 1D and 2D NMR proving the formation of the exclusion-type complex with α-CD and pseudorotaxane inclusion-type complexes with β-, and γ-CDs. UV–vis titrations at pH 7.4 gave association constants for the obtained complexes. The stability of the complexes increases in the series: α-CD/PTX < γ-CD/PTX << β-CD/PTX. The association of PTX with a monomer cyclodextrin equivalent – methyl α-D-glucopyranoside – was investigated for a deeper understanding of the type of host–guest interactions. Solid state studies of PTX/CDs were performed using FTIR–ATR and Raman spectroscopy techniques.

## Introduction

**Figure 1:**
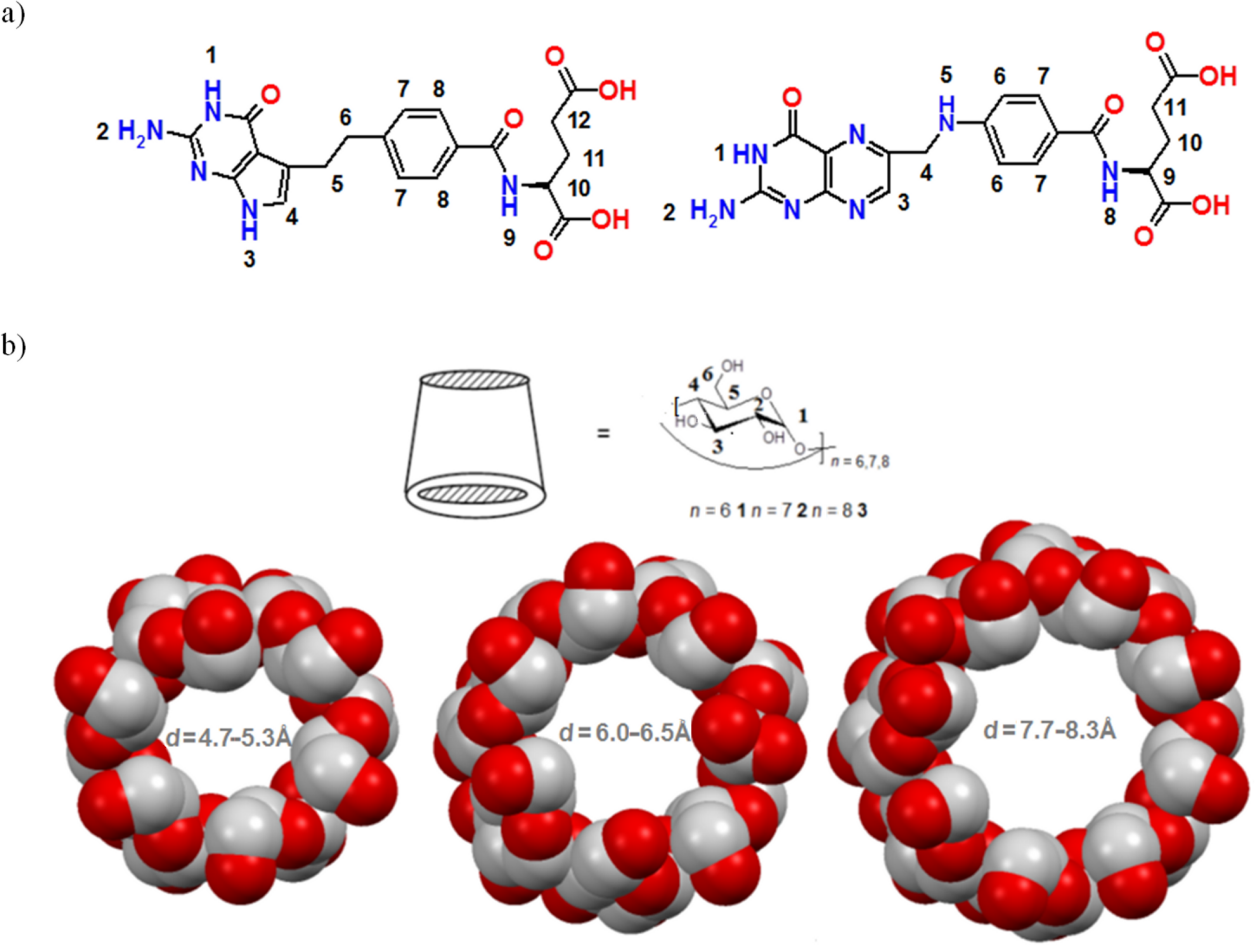
Molecular structures with atom numbering of the guest (a: from left to right): pemetrexed (PTX) and folic acid (FA), and host molecules with increasing diameter of macrocyclic cavity (b: from left to right: α-, β- and γ-CDs).

Herein, supramolecular host–guest complexes of the potent folic acid inhibitor pemetrexed (PTX, Figure 1a) with native cyclodextrins (α-, β- and γ-CDs, Figure 1b) in the gas phase (MS), in solution (NMR, UV–vis) and in the solid state (Raman, FTIR–ATR) were studied.

Native CDs are water-soluble cyclic oligosaccharides, products of starch degradation, which contain six (α-CD), seven (β-CD) or eight glucopyranose units (γ-CD), accordingly, linked by α-1,4-glycosidic bonds (Figure 1b). They are toroid-like shaped and possess a cone-like hydrophobic cavity of various sizes (4.7–8.3 Å) into which distinct compounds may enter and form inclusion complexes, an ability that is thoroughly used by the pharmaceutical industry for the formation of drug delivery systems [1–5]. The formation of an inclusion complex often results in a favorable change of pharmacokinetic properties of the guest molecule, such as water solubility, bioavailability and stability [6–7]. In particular, many important drugs in cancer treatment are often toxic and/or sparingly soluble or even insoluble in water. Among other methods, reversible derivatization of these drugs into CD complexes is a highly attractive way to overcome these unwanted features [8]. One of such chemotherapeutic drugs is pemetrexed (PTX), developed initially in 2004 for the treatment of non-small lung cancer [9]. This type of cancer is the most commonly appearing cancer in the world [10] and its treatment still presents a challenge for medicinal and pharmaceutical studies, involving the search for new molecules acting as folate inhibitors [11–12], as well as their recognition mechanisms [13–14]. The molecular structure of PTX closely resembles folic acid (FA, Figure 1a), and hence, it belongs to the class of folate antimetabolites (antifolates) that act by inhibiting intracellular folate-using enzymes. PTX enters the cell through the reduced folate carrier and transforms into a polyglutamated intermediate which is able to inhibit three enzymes: dihydrofolate reductase (DHFR), thymidylate synthase (TS) and glycinamide ribonucleotide formyl transferase (GARFT) [15]. Despite these advantageous properties, PTX exhibits some severe toxicity.

In this work, the formation of the complexes of all three native CDs with PTX was studied in solution by the means of ^1^H NMR which gave information about the conformation of the guest molecule as well as its possible interactions with host CDs. All experiments were done in D_2_O, and also in DMSO-*d*_6_ which enabled tracking of the changes of chemical shifts of acidic protons. The architecture of the formed complexes was then studied by 2D ROESY NMR. UV–vis titration experiments in phosphate buffered saline (PBS, pH 7.4) were conducted for evaluation of association constants (*K*_as_). Association of PTX with a monomer CD equivalent – methyl α-D-glucopyranoside – was investigated to fully understand the type of interactions between host and guest molecules. Additionally, the CDs/PTX complexes were examined in terms of complex stability in gas-phase collision induced dissociation (CID) experiments using mass spectrometry (MS). This approach provides insight into the intrinsic features of these noncovalently associated molecules, i.e., without the presence, and hence possible interference, of the solvent molecules. The gas-phase stability of such a complex, depending on the nature of interactions occurring between molecules within a complex, may reflect the solution binding behavior, in particular when polar interactions greatly contribute to the overall binding energy. Recent results concerning the studies of association modes between FA and CDs clearly showed that, although the overall gas-phase stabilities do not reflect the solution phase association constants, they are helpful in structural differentiation between inclusion and exclusion complexes [16]. The complexes in which a FA guest is trapped in the central cavity of CD in a form of pseudorotaxane structure are supposed to be substantially more stable than complexes with a ligand being associated with the CD’s surface or partially allied with CD’s internal space. In the solid state two complementary methods – FTIR–ATR and Raman were used for describing the way of PTX-CD interactions. The IR spectroscopy in the region characteristic for δ-HOH bending of water molecules attached to CDs (1600–1700 cm^−1^) is usually employed for tracking of the inclusion complex formation, however, Raman spectra of this region are far more clearer, since no interfering bands from CDs are present. In addition, this region of the Raman spectra is characteristic for C=C vibrations and provides clear evidence of the inclusion of the guest molecule, in particular aromatic one, within the CD cavity, and hence unequivocal identification of guest–host interactions is possible [17–18]. Any changes in the band position as well as increasing or decreasing of its intensity indicate complex formation. In that sense Raman spectroscopy emerges as an important technique for studying host–guest interactions, in particular for verifying if the guest molecule is located inside the CD cavity.

The present study serves two purposes. The first is studying of the process of formation of the host–guest complexes of all three native CDs with PTX, an antifolate exhibiting considerable toxicity and their detailed characterization in the gas phase, in solution and in the solid state. Critical analysis of the data obtained for native CDs/PTX as well as for complexes with FA, a guest molecule structurally related to PTX would help in mapping host–guest interactions between native CDs and other, biologically important, structurally related molecules. The experiments focused on association of PTX with monomer cyclodextrin equivalent – methyl α-D-glucopyranoside – were designed to provide additional information about the type of interactions between host and guest molecules.

The second aim of this work is to demonstrate the importance of comparison of the data obtained from the gas phase (without the influence of the environment) with the data obtained for solution and solid state (strongly influenced by the molecule’s environment) for deeper understanding of the non-covalent interactions responsible for the formation and stabilization of such supramolecular complexes.

## Results and Discussion

### Studies of PTX complexes in the gas phase. Intrinsic stability of PTX/CDs complexes

Initially, the noncovalent CDs/PTX complexes were subjected to mass spectrometry analysis to investigate the binding mode, stoichiometry, and relative gas-phase stabilities of these noncovalent complexes. From the previous studies it is known that collision-induced dissociation (CID) experiments may be helpful in distinguishing between exclusion and inclusion complexes via comparison of the relative complex stabilities. For example, inclusion complexes of folic acid (FA) with β-CD and γ-CD are far more stable than exclusion associate of FA and α-CD [16]. From collisional experiments we have also learnt that augmented stability of both inclusion complexes is mainly due to the increase of electrostatic forces and hydrogen bonds between FA and β- or γ-CD in the gas phase. The hydrophobic interactions which are important stabilizing forces in solution are significantly diminished in the gas phase and do not manifest in the collisional experiments as it was concluded by the observation of increased gas-phase stability of γ-CD/FA complex comparing to its noncovalent interaction strength in solution. The increased influence of hydrophobic effects on the overall stabilities of CDs/FA complexes was observed in experiments in which a mass spectrometer was treated as a detector for quantitative analysis of CDs/FA complexes. Only these experiments enabled the solution-like order of stability to be obtained [16]. Similarly to the CDs/FA complexes, PTX with α-CD, β-CD, and γ-CD forms stable noncovalent associates (1:1 host–guest) in the gas phase, which are detected as doubly negatively charged ions ([CD + PTX − 2H]^2−^) in the negative-ESI mass spectra (see Supporting Information File 1: Figure S1a–c). Other stoichiometries (2:1, 3:1, host–guest) were also detected in the upper *m*/*z* range of the mass spectra. The intensities of these associates were very low (no exceeded 10% of the intensity of the 1:1 complexes). The fragmentations of the 2:1 complexes are multipathways and contain the loss of fragments corresponding to the following ions: [2CD − H]^−^, [PTX − H]^−^, [PTX + CD − H]^−^, [CD − H]^−^, and [PTX + CD − 2H]^2−^. The main complex disintegration process, comprising separation into [2CD − H]^−^ and [PTX − H]^−^, occurs at very low collision energy. This observation suggests that the PTX molecule is loosely attached to the surface of two (or more) closely bonded CDs, thus forming weakly bound exclusion-type associates.

All 1:1 CDs/PTX complexes follow the same dissociation pathway, i.e., they separate into singly charged anions of PTX and CD (the CID spectra are available in Supporting Information File 1, Figure S2a–c). The exclusive fragmentation pathway of studied complexes into two components suggests that PTX is quite loosely connected with the CD comparing to a typical rotaxane complex for which the main fragmentations, occurring at substantial high collision energies, are these resulting from covalent bond cleavages [19]. The loosely bonded CDs/PTX complexes therefore correspond to a pseudorotaxane inclusion complex in which PTX is threaded through the CD’s cavity or other type of exclusion aggregate. To distinguish between these two types of complexes their relative stabilities were determined by CID experiments.

**Figure 2:**
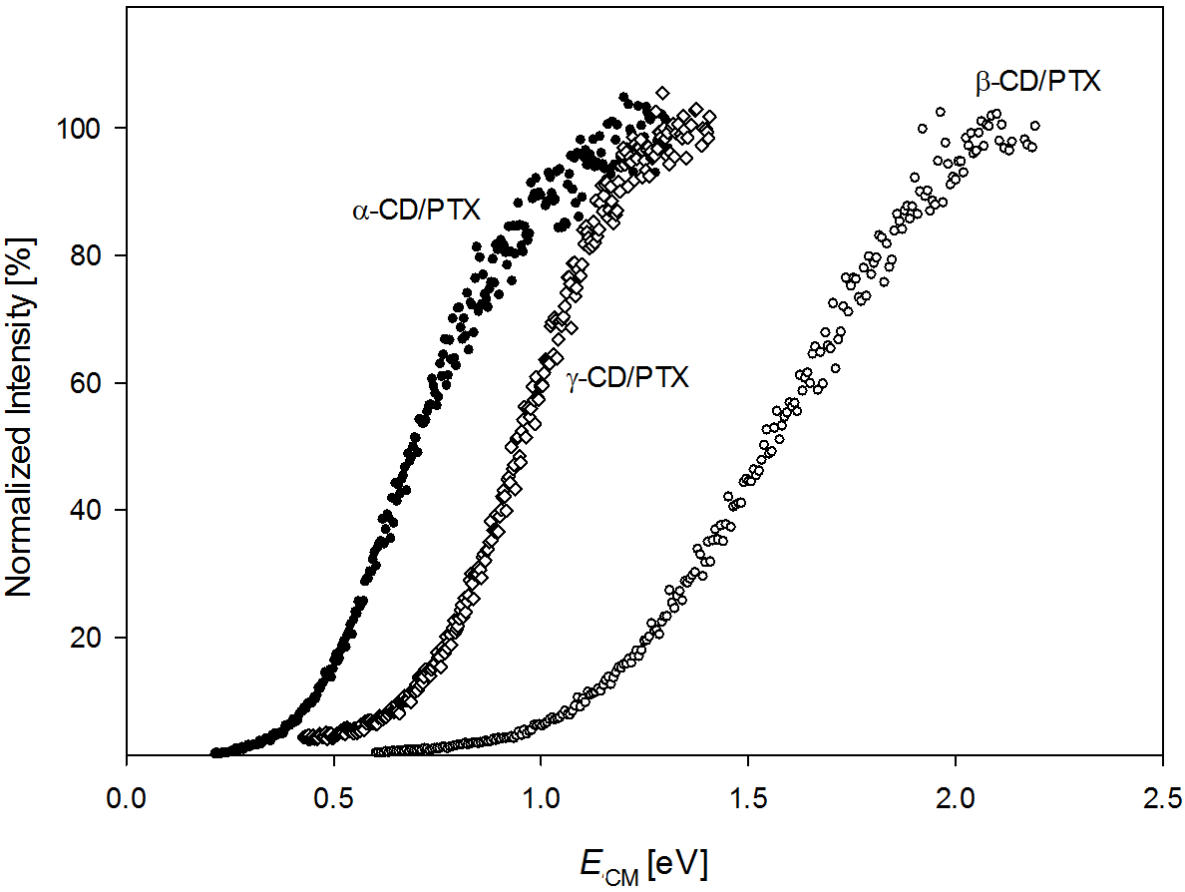
Dissociation efficiency curves of three CDs/PTX complexes. The ions were collided with nitrogen (pressure ca. 2 × 10^−5^ Torr) at various collision energies. The y axis represents the normalized intensity of the PTX anion ([PTX − H]^−^) in proportion to the intensity of a complex ([CD + PTX − H]^2−^).

The collision energies for half-dissociation of CDs/PTX complexes (CE_50%_) as a measure of complex stabilities were established based on the dissociation efficiency curves (plots of normalized intensity of complexes vs center of mass collision energy under single collision conditions – Figure 2). The values of CE_50%_ for complexes of PTX with α-, β-, and γ-CD are 0.73 eV, 1.56 eV, and 0.94 eV, respectively. These differences in stabilities of CDs/PTX allowed us to propose topologies for analyzed complexes based on the earlier evaluated ones for the CDs/FA structure–stability relationship [16,20].

Similar to CDs/FA complexes, the most stable complex of PTX with β-CD corresponds to a pseudorotaxane structure, whereas the least stable α-CD/PTX coincides with an exclusion complex. In contrast to the γ-CD/FA complex, the stability of the γ-CD/PTX complex given by the CE_50%_ is just between the values determined for complexes of PTX with α- and β-CD. Thus, the structural details of the γ-CD/PTX associate remain unclear and direct assignment to inclusion- or exclusion-type complex is not possible based on the exclusive CID experiments.

### Studies of PTX complexes in solution

#### Evaluation of association constants of native CDs with PTX

The association constants (*K*_as_) were evaluated by UV–vis titrations at pH 7.4 (in phosphate buffered saline, PBS). PTX shows two absorption maxima at 226 and 245 nm (for absorption spectra of PTX upon titration with β-CD see Figure S3 in Supporting Information File 1). Upon titration, to avoid dilution effects and the influence of a possible guest aggregation, the concentration of the guest molecule was kept constant. With the increase of the β-CD concentration the absorbance at the same wavelength decreased, while for γ-CD increased. For evaluation of association constants (*K*_as_) the obtained data were fitted with the HypSpec program, which enables global fitting of absorption data at all wavelengths. The corresponding titration curves for β-CD/PTX and γ-CD/PTX were consistent with a 1:1 host–guest binding model (for titration plots see: Figures S4 and S5 in Supporting Information File 1, for *K*_as_ values see Table 1). The changes observed for α-CD were the smallest resulting in the lowest association constant (*K*_as_ = 4), suggesting very low affinity of α-CD towards PTX. It is worth to notice that a similar complex stability (β-CD/PTX >> γ-CD/PTX > α-CD/PTX) was derived from MS experiments in the gas phase.

**Table 1:** The values of binding constants *K_as_* [M^−1^] for PTX with native CDs measured at pH 7.4.

	α-CD	β-CD	γ-CD

PTX	4	226 ± 4	32 ± 0.1

Similarly, for the CDs/FA complexes, β-CD produced the most stable complex, whereas α-CD formed the least stable exclusion-type complex. In the case of γ-CD host, for both FA and PTX guests weak interactions were observed. It is also consistent with literature data regarding another structurally related antifolate – methotrexate, where β-CD formed stable complexes [21–22], whereas for γ-CD weak binding was observed [21].

### Studies of native CDs/PTX complexes in solution by the means of ^1^H NMR and 2D ROESY NMR

The supramolecular complexes of PTX with native CDs were studied in solution by the means of ^1^H NMR and 2D ROESY NMR (Supporting Information File 1, Figures S6–S23).

**Figure 3:**
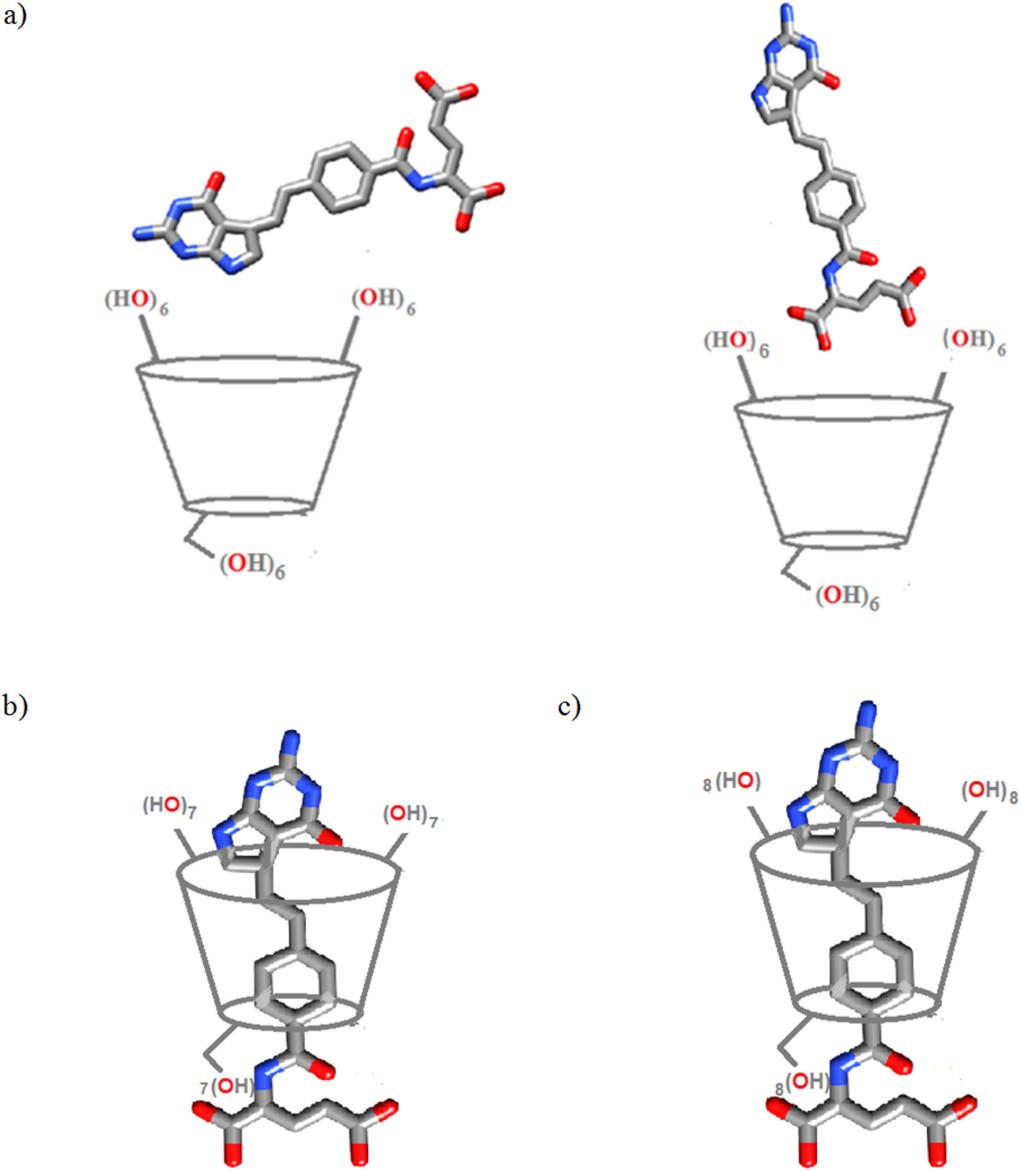
The proposed molecular structures of PTX complexes with α-CD (a: exclusion-type), β-CD (b: rotaxane-like), and γ-CD (c: rotaxane-like); for α-CD/PTX two equally probable conformations are shown.

**α-CD/PTX and its comparison with α-CD/FA:** In the ^1^H NMR spectra of an equimolar mixture of α**-**CD and PTX in D_2_O only marginal changes in chemical shifts (Δδ) of α**-**CD host are observed. The most pronounced shift appears for CD#H4 directed outside of the host cavity (Δδ = 0.02 ppm). This suggests that the PTX guest is too large to penetrate into the α**-**CD cavity; hence the stability of the α**-**CD/PTX associate should be low. This assumption was fully confirmed by the results of the 2D ROESY NMR experiments. The absence of any cross-peaks between α**-**CD and PTX molecules, both in 1:1 and 1:16 molar ratio mixtures, ruled out the existence of an inclusion complex. Nevertheless, some pronounced changes for the protons belonging to the PTX molecule are observed (Δδ = 0.03–0.34 ppm, see Supporting Information File 1, Figures S6, S12, S13 and Table S1). This may suggest that carboxylate groups of the PTX dianion are involved in the interaction with the hydrophilic rims of α-CD molecule. In order to test this possibility we decided to measure the ^1^H NMR spectrum of an equimolar mixture of PTX with methyl α-D-glucopyranoside, the monomer equivalent of studied CDs (Supporting Information File 1, Figure S7). We observed a similar interaction pattern as in the case of α-CD/PTX, i.e., PTX#H4, PTX#H7, PTX#H8 shift considerably downfield after addition of methyl α-glucopyranoside (Δδ = 0.04–0.07 ppm, Supporting Information File 1, Figure S4, Table S2). This strongly supports the assumption that the PTX dianion interacts with hydroxy groups of the sugar moieties, and this phenomenon was already highlighted in the literature [23–24]. Keeping in mind that signals of acidic protons are not visible in D_2_O due to the fast substitution by deuterium atoms we performed 1D NMR measurements in DMSO-*d*_6_ (see Figures S15–S17, S18, S23 and Tables S5 and S6 in Supporting Information File 1). From these experiments it can be clearly seen that pyrrolic proton PTX#H4 interacts with hydroxy groups of the wider rim of α-CD (CD#OH2 and CD#OH3). Changes upon complexation are also seen for PTX#H1 while the chemical shift of amidic proton PTX#H2 does not change at all, suggesting the lack of any interactions with the host molecule. In addition, the obtained data suggests that the free PTX molecule is engaged in strong H-bonds between the carboxylate donor –NH group, which are gradually broken. Overall all the data obtained from 1D and 2D NMR experiments suggests formation of exclusion-type associate of two equally probable conformations depicted in Figure 3a.

Moreover, from NMR studies one can recognize that PTX interacts in a similar manner as FA [16,20] with the α-CD host. Neither of the guest molecules is included in the cavity of the host but they both interact weakly with the outside shell of the host CD. There are, however, some important differences. The changes of chemical shifts for the guest molecule in regard to FA appear only for three protons (aliphatic FA#H4 protons and phenolic FA#H6 and FA#H7) while changes for PTX appear in the same region but additionally for the belonging to the bicyclic heteroaromatic proton PTX#H4. It means that either this part of the guest molecule finds itself closer than FA to the CD molecule or/and the change in the overall conformation is more substantial.

**β-CD/PTX and its comparison with a β-CD/FA complex:** Analysis of the ^1^H NMR spectrum of an equimolar mixture of β-CD and PTX in D_2_O indicates that changes in chemical shifts of both host and guest protons (Figures S8, S12 and S13, and Table S3 in Supporting Information File 1).

The most pronounced changes appear for protons of PTX#H4, PTX#H5, PTX#H6, and PTX#H7 (Δδ being 0.09, 0.06, 0.06, and 0.10 ppm, respectively; see Supporting Information File 1, Table S3). The changes in chemical shifts of β-CD protons appear for all protons (Table S3), in particular for inner cavity H3 and H5 protons (0.12 and 0.08 ppm, accordingly), which demonstrates that the guest molecule is included in the β-CD cavity.

**Figure 4:**
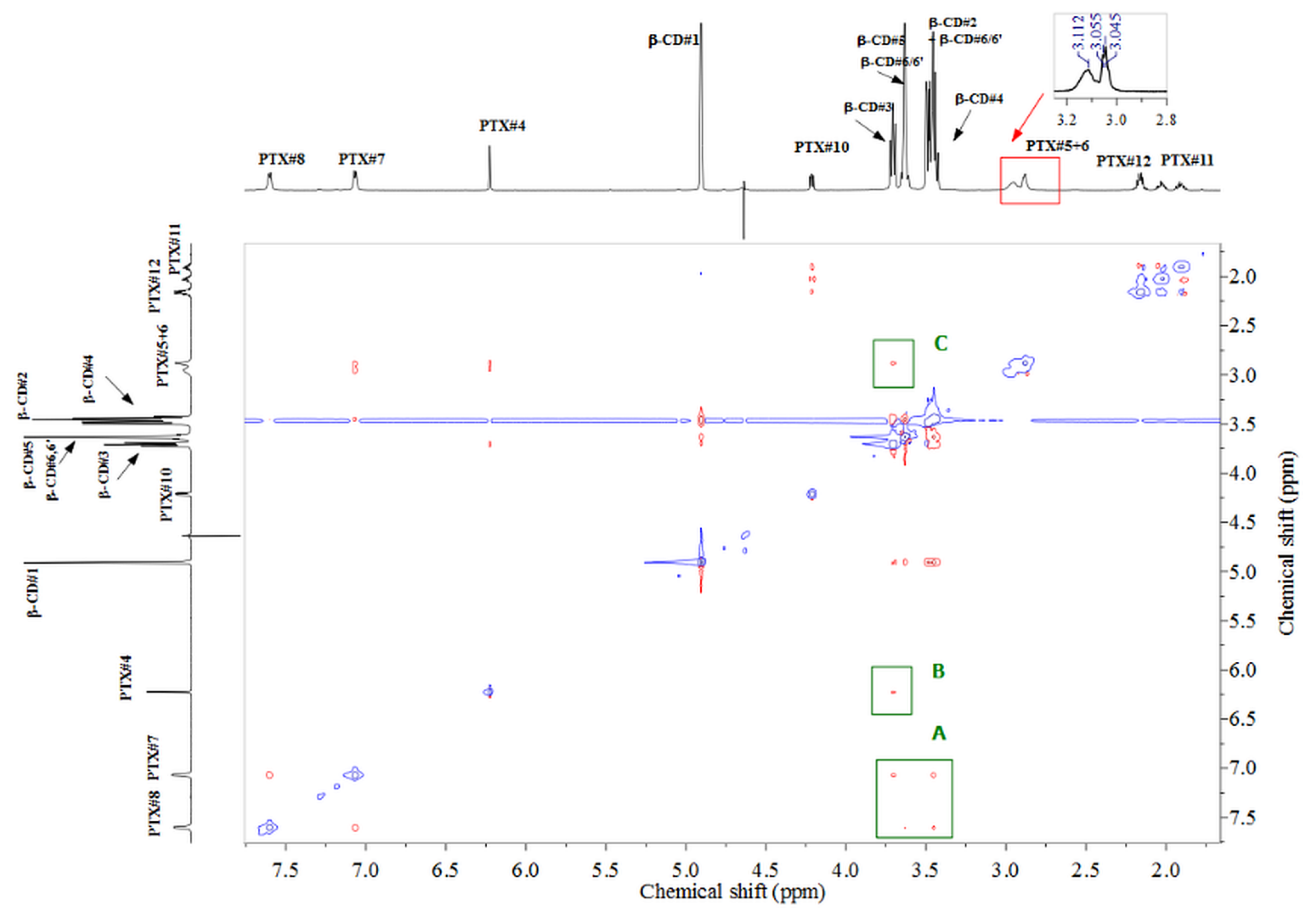
2D ROESY NMR spectrum of an equimolar mixture of β-CD and PTX in D_2_O at 298 K. Inset: splitting of the bridging ethylene protons after addition of β-CD.

In addition, splitting of the signal assigned to bridging ethylenic protons PTX#5 and H#6 is observed (see Figure 4 left inset and Supporting Information File 1, Figure S14) clearly indicates hindrance of rotation, due to a partial inclusion inside the host cavity. This splitting was not observed for α-CD/PTX exclusion-type associate.

Likewise, the 2D ROESY NMR spectrum of β-CD/PTX complex (Figure 4) displays clear cross-peaks between accordingly phenyl (H7 and H8, region A), pyrrole (H4, region B), and aliphatic linker (H5-H6, region C) protons of the PTX guest with the C–H ring protons of the β-CD host (H2, H3, H4 and H5). Protons H3 and H5 of β-CD which are directed inside the cavity are responsible for the formation of favorable hydrophobic interactions with the guest molecule. Similarly, H2 and H4 protons which are directed outwards the cavity may also interact, plausibly indirectly via OH hydrogen bonds, with guest molecules located outside the cavity. The obtained results strongly support the assumption that not a whole PTX molecule is encapsulated inside the β-CD cavity, and, instead, the complex is formed through inclusion of a major part of aromatic parts of the molecule (both bicyclic heteroaromatic ring and phenolic ring), whereas aliphatic chain with carboxylic functionalities is dangling outside the host molecule forming a rotaxane-like inclusion compound (Figure 3b).

For more detailed information on the behavior of acidic protons of the guest molecule 1D measurements in DMSO-*d*_6_ were performed (see Supporting Information File 1, Figures S15, S16, S18, S21, S23, and Tables S5 and S6). The pyrrolic proton PTX#H4 interacts with CD#OH2 and CD#OH3 hydroxy groups of the wider rim of β-CD which support the up-and-down position of the guest molecule within the β-CD cavity (as depicted in Figure 3b). Changes upon the complexation are also seen for PTX#H1 while the chemical shift of amidic proton PTX#H2 does not change.

2D ROESY NMR of β-CD/PTX (Figure 4) closely resembles 2D NMR spectrum of β-CD/FA [20] suggesting that for both guests a similar mode of complexation is taking place. Comparable changes are also observed in ^1^H NMR after complexation of the guest compounds with the changes being larger for the PTX molecule. Moreover, a quite substantial change in chemical shift upon complexation is observed for the PTX#H4 proton of the bicyclic part of the PTX molecule (0.09 ppm), whereas for FA the corresponding change of the FA#H3 proton equals only 0.05 ppm. Overall all the changes observed for the complexation of PTX are larger than for FA, which suggests stronger binding and/or much pronounced conformation change for PTX than for FA guest.

**Figure 5:**
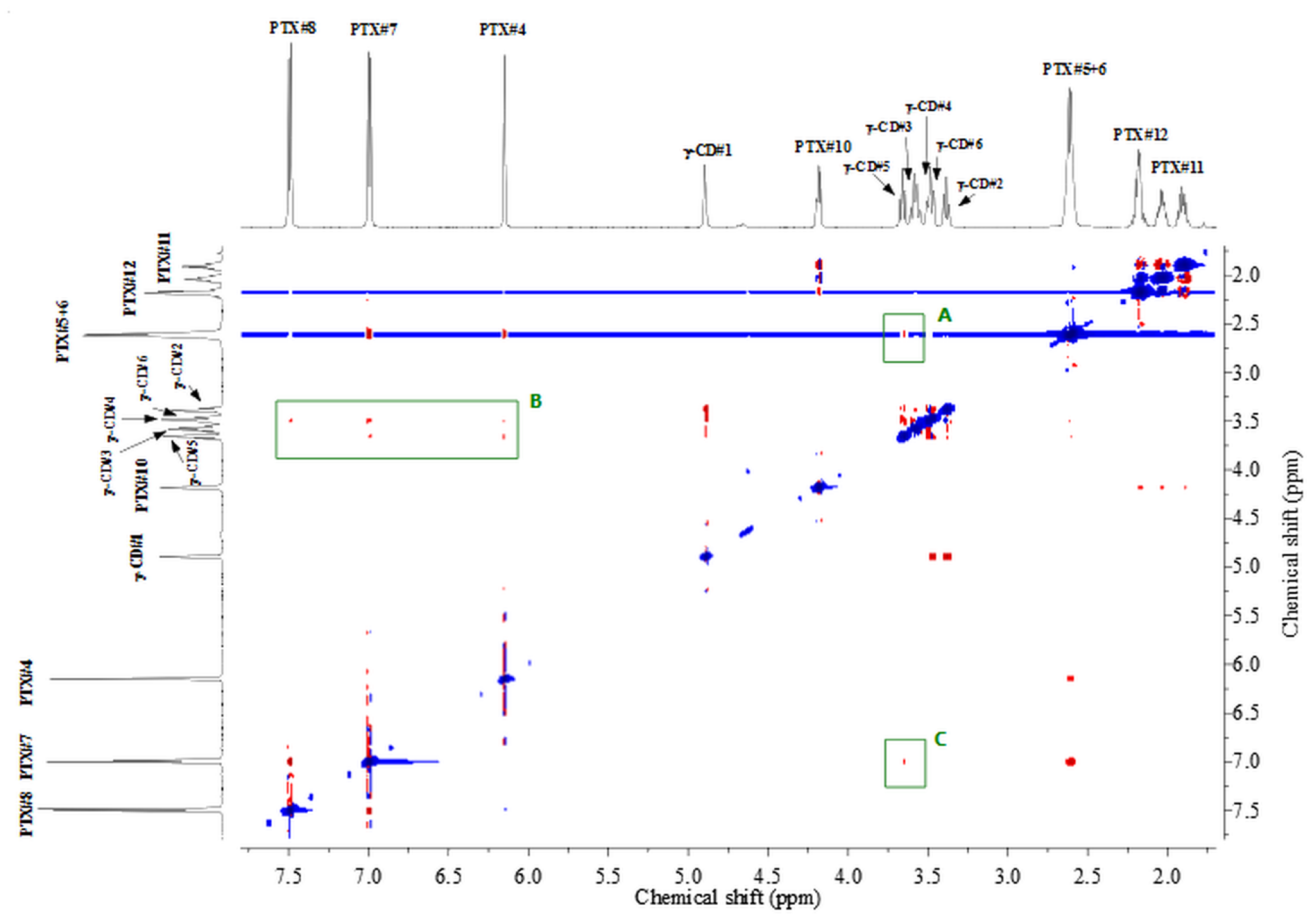
2D ROESY NMR spectra of a mixture of γ-CD with a 16-fold molar excess of PTX added in D_2_O at 298 K.

**γ-CD/PTX and its comparison with a γ-CD/FA complex:** The analysis of the ^1^H NMR spectrum of an equimolar mixture of γ-CD with PTX in D_2_O clearly indicates that there are only marginal changes in the chemical shifts of γ-CD host (Table S4, Supporting Information File 1). Nevertheless, slight upfield shifts are observed for intracavity protons H3 and H5 which suggest guest inclusion. Similarly to associates with α- and β-CDs, there are significant changes for the protons belonging to the PTX molecule, which further supports that PTX is engaged in hydrogen bonds with CD’s hydroxy groups (Table S4 and Figures S9, S12 and S13, Supporting Information File 1). In order to establish the type of interactions between these molecules we have carried out 2D ROESY experiments (1:1 host–guest ratio, see Figure S11, Supporting Information File 1). These experiments suggest very weak interactions between the CD host and the PTX guest. For weak complexes the clearer picture of the associate’s architecture may be obtained when experiments are performed with an excess amount of the guest molecule. In our case, 2D ROESY NMR (1:16 γ-CD/PTX molar ratio) showed an additional clear cross-peak between PTX#H7 and the inner cavity H5 proton of γ-CD (Figure 5).

As in the case of β-CD/PTX, splitting of the bridging ethylenic signal was observed (PTX#5 and H#6, Figure S14, Supporting Information File 1). Based on these results we assumed that guest molecule penetrates γ-CD from the wider rim and is inserted into the macrocyclic cavity. To further confirm these findings 1D measurements in DMSO-*d*_6_ were performed (see Supporting Information File 1, Figures S15, S16, S19, S22, S23, and Tables S5, S6). The pyrrolic proton PTX#H4 interacts with the CD#OH2 and CD#OH3 hydroxy groups of the wider rim of γ-CD which suggests the up-and-down position of the gust molecule inside the complex (see Figure 3c). The obtained results are similar as for the analogous γ-CD/FA complex, both regarding the architecture and the strength of the obtained inclusion compounds.

### Studies of PTX complexes in the solid state

#### FTIR–ATR

The FTIR spectra of CDs/PTX complexes may be divided into three regions (Supporting Information File 1, Figure S25, and Table S8; for spectra of pure native CDs see Figure S24 and Table S7). The first region in the range of 800 to 950 cm^−1^ belongs to the deformation vibrations of the C–H bonds and pulsation vibration of the glucopyranose ring of CD. The second region in the range of 1050 to 1100 cm^−1^ is characteristic for the valence vibrations of the C–O stretching bands in the ether and hydroxy groups of CDs. Third region in the range of 1430 to 1650 cm^−1^ is attributed to C=C stretching vibrations of the guest molecule. For pure CDs only one diagnostic vibration exists in that region (1642, 1647 and 1643 cm^−1^ for α-, β- and γ-CD, respectively) which reflects the δ-HOH bending of water molecules attached to CDs [25–26].

**Figure 6:**
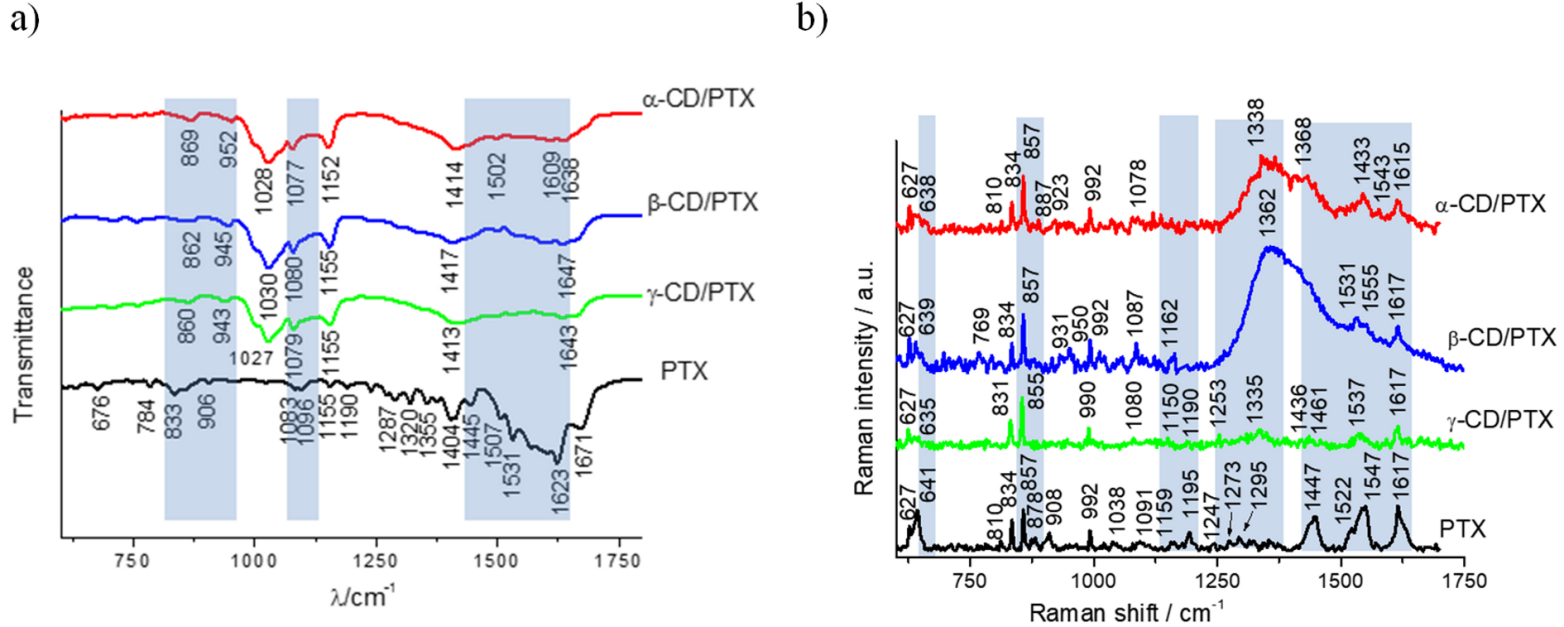
a) FTIR–ATR and b) Raman spectra of PTX together with its complexes with α-CD, β-CD and γ-CD.

In Figure 6a the FTIR spectra of PTX and its complexes with native CDs are shown. The vibration bands at 833 and 906 cm^−1^ for the PTX molecule show shifting in the α-CD/PTX complex (869, 952 cm^−1^) while for β-CD/PTX and γ-CD/PTX band shifting is accompanied by the decrease of intensity.

For the free PTX molecule two small intensity vibrations at 1083 cm^−1^ and 1096 cm^−1^ exist, which are largely shifted to the smaller wavenumbers in all of its associates CD associates (1028, 1078 cm^−1^ in α-CD/PTX; 1030, 1080 cm^−1^ in β-CD/PTX; 1027, 1079 cm^−1^ in γ-CD/PTX). In addition, for the PTX molecule few bands are observed in the range of 1430 to 1650 cm^−1^ (1445, 1507, 1537, 1569, 1603 cm^−1^). For the α-CD/PTX complex due to interactions between host and guest, new bands characterized by small intensities appeared at 1502, 1540, 1566, 1609, 1638 cm^−1^; at 1500, 1602, 1633 cm^−1^ for β-CD/PTX; and at 1496, 1528, 1632, 1663 cm^−1^ for γ-CD/PTX. All these additional bands indicate host–guest interactions between PTX and the host CD molecules.

The FTIR spectra of CDs/PTX complexes show shifting and decreasing of the band intensities, especially in the region characteristic for C=C band vibrations, which clearly indicate strong interaction between these molecules. Such behavior allows for the assumption that PTX guest is bound from outside part of the α-CD host, thus forming exclusion-type complex, and inclusion of PTX into β-CD and γ-CD cavities.

#### Raman spectroscopy of CD/PTX complexes

It is known that the Raman spectrum of the physical mixture of the CD-host and guest molecules shows spectral features almost identical with guest molecule [27–28]. That is due to the usually very small intensity of observed Raman bands of the native CD in comparison to the bands of the guest molecule [29]. In contrary, the spectrum of the host–guest complexes, where due to molecular interactions some changes in Raman spectra would be observed (shifting or decreasing of the peak intensity or broadening of characteristic bands) provide information about host–guest interactions in the formed complexes. As an example, such behavior for aromatic ring bands, besides the existence of the guest–host interactions, would suggest that the aromatic ring of the guest moiety is included in the CD cavity. Moreover, the spectral region characteristic for vibrations of aromatic bands (1550–1800 cm^−1^) deserves special attention since this area is completely free of interfering bands from the CD hosts.

For CDs/PTX host–guest complexes in Raman spectra, similar to the FTIR spectra, five most prominent regions indicating interactions within CDs and PTX molecules are observed (Figure 6b and Supporting Information File 1, Table S10; for spectra of pure native CDs see Figure S25 and Table S9). The 641 cm^−1^ band is characteristic for PTX and for CD/PTX complexes (638, 639 and 635 cm^−1^ for α-, β-, and γ-CD, respectively). A similar behavior can be noticed in the case of two other PTX bands at 810 cm^−1^ and at 878 cm^−1^. The first of these vibrations occurs in the Raman spectrum of two CD/PTX complexes. For α-CD/PTX this band appears at 810 cm^−1^, it is slightly shifted for β-CD/PTX (769 cm^−1^) and is not observed at all for the γ-CD/PTX complex. The second band observed for PTX (878 cm^−1^) appears only for α-CD/PTX (887 cm^−1^). Another region characteristic for C=C–H and =C–C= vibrations is observed for free PTX (1159 cm^−1^) and the β-CD/PTX complex (1162 cm^−1^). Shifting indicates interaction between PTX and β-CD. This type of band is also observed for all native CDs (1140 cm^−1^ for α-CD, 1132 and 1150 cm^−1^ for β-CD, 1131 cm^−1^ for γ-CD, see Supporting Information File 1, Figure S25). Moreover, it is worth mentioning that some additional, very low intensity bands at 1247, 1273, 1295 cm^−1^ which are observed in the Raman spectrum of PTX are absent in Raman spectra of its two derivatives (i.e., α-CD/PTX, β-CD/PTX), while for the γ-CD/PTX complex a very weak peak at 1253 cm^−1^ is observed. This specifies different host–guest interactions with the CD surfaces for α-CD/PTX, and β-CD/PTX complexes and the characteristic ones for the γ-CD/PTX. Such behavior of Raman vibrations indicates that the PTX guest molecule is located inside the γ-CD cavity but in different way than in β-CD. In the spectral region characteristic to the C=C band vibrations the Raman spectrum of free PTX exhibits the most characteristic bands at 1447, 1547 (with small shoulder at 1522 cm^−1^) and 1617 cm^−1^. For α-CD/PTX complex two bands at 1543 cm^−1^ and 1615 cm^−1^ are observed. These, very slight shifting in the band positions, indicate the existence of PTX molecules outside α-CD structures and not inside the cyclodextrin cage. For β-CD/PTX three vibrations at 1531, 1555 and 1617 cm^−1^ and for the γ-CD/PTX complex two vibrations at 1537 and 1617 cm^−1^ are observed. A small host–guest interaction, in the form of band shifts is evident in all obtained spectra of CD/PTX complexes when compared with the spectrum of the free PTX. However, for α-CD/PTX, this shifting is the smallest one, and is more pronounced in Raman spectra of γ-CD/PTX and β-CD/PTX. To summarize: Raman spectra indicate formation of an exclusion-type complex of α-CD/PTX and inclusion-type complexes of β-CD/PTX and γ-CD/PTX. However, the interactions within these latter two complexes are different.

## Conclusion

In the present work the complexation of PTX with native CDs, namely α-CD, β-CD, and γ-CD, was studied in the gas phase (MS), in solution (NMR), and in the solid state (Raman, FTIR–ATR). ESIMS proved the formation of 1:1 associates for all studied cases [M(α-CD/PTX − 2H)^2−^ = 699.1; M(β-CD/PTX − 2H)^2−^ = 780.1; M(γ-CD/PTX − 2H)^2−^ = 861.1]. We found that cyclodextrin with the smallest cavity, α-CD, forms exclusion type complex (as seen from 1D NMR in D_2_O and DMSO-*d*_6_ and 2D ROESY NMR experiments). β-CD and γ-CD form rotaxane-like structures where CD host is threaded over the PTX guest, as it is clearly seen from 2D ROESY experiments. In addition, splitting of the diagnostic NMR signal assigned to bridging PTX ethylenic protons was observed for β-CD/PTX and γ-CD/PTX, but not for α-CD/PTX, which further supports the postulated binding mode of CDs with the PTX guest. Moreover, information obtained from NMR experiments performed for PTX mixtures with CDs simplest analogue – methyl α-D-glucopyranoside showed that PTX dianion interacts with hydroxy groups of sugar moieties. This additionally proved that unusually large changes of chemical shifts for PTX upon addition of α-CD were caused by carboxyl anion interactions with hydroxy groups of the α-CD. The highest affinity for binding of PTX at pH 7.4 (physiological buffer, PBS) was observed for β-CD (*K*_a_ = 226 ± 4 M^−1^). γ-CD formed complexes characterized by lower association constant *K*_a_ = 32 ± 0.1 M^−1^, whereas α-CD host was shown to have very low affinity toward PTX (*K*_a_ = 4 M^−1^). Findings from FTIR–ATR and Raman experiments demonstrate that the region of C=C bond vibrations of the guest is an excellent indicator of complex formation. The observed changes in this band (position and intensity), together with the information about possible appearance of new bands, may allow for the classification of which particular host–guest interaction is taking place in each analyzed case. Finally, we showed that results from gas-phase, solution and solid state studies are complementary to each other proving that such comprehensive study employing different techniques (NMR, CID-MS, FTIR–ATR and Raman spectroscopy) allows for a deeper and clearer understanding of the molecular recognition of native CDs.

## Experimental

### Materials

FA (≥97%) was obtained from Sigma-Aldrich and used as disodium salt. PTX-Na_2_ (≥98%) was obtained from ChemScene (USA). α-, β-, and γ-CDs of standard grade were obtained from Cyclolab (Hungary) and used as received. For aqueous solutions HPLC purity grade H_2_O and D_2_O (99.9% D) were used. D_2_O and DMSO-*d*_6_ was purchased from Cambridge Isotope Laboratories.

### Preparation of samples by solvent evaporation method

To the solution of PTX sodium salt in water (47.1 mg, 1 equiv per 2 mL) or FA sodium salt in water (66.2 mg, 1 equiv per 5 mL H_2_O) a solution of α-CD in water (97.2 mg, 1 equiv per 8 mL H_2_O) was added and thus 1:1 obtained solution was stirred at rt for 48 h. The solvent was evaporated and the resulting solid was dried under vacuum. The procedure was repeated for β- and γ-CDs using: 113.5 mg of β-CD, 129.7 mg of γ-CD for PTX and 92.5 mg of β-CD in 5.6 mL H_2_O, 232 mg of γ-CD in 1.2 mL H_2_O for FA, respectively. For α- and γ-CDs additional samples with 1:16 host:guest molar ratio were prepared.

### Characterization of the native CDs/PTX complexes

#### Mass spectrometry. Collision-induced dissociation (CID) MS experiments

The basic mass spectrometry spectra (Q1) and collision dissociation spectra were recorded on a 4000-QTRAP triple quadrupole mass spectrometer (Applied Biosystems). The mass spectrometer was equipped with a TurboIonSprayTM electrospray ion source operated in the standard negative ESI mode, i.e., without additional drying gas. The three solutions containing PTX sodium salt (0.04 mM) and α-CD, β-CD, and γ-CD (0.04 mM) were prepared in water/methanol (1:1 v/v). Analyte solutions were introduced into the ion source with a syringing pump at a flow rate of 10 µL/min. The ion source parameters were optimized to obtain the highest possible abundance of the complex anions and were adjusted as follows: ion-spray voltage (IS) −3.5 kV, declustering potential (DP) −15 V, entrance potential (EP) −10 V. In CID experiments, nitrogen was used as the collision gas and the collision energy varied in the range of −5 eV to −50 eV. The collision-induced dissociations of CDs/PTX complexes ([CD + PTX − 2H]^2−^) into PTX ([PTX − H]^−^) were monitored using MRM mode. The intensities of the MRM peak at various collision energies were used to obtain dissociation efficiency curves (plots of normalized intensity of PTX anion in proportion to the intensities of the complexes vs the center of mass collision energy under single collision conditions). The collision energy for half-dissociation of a complex (CE_50%_) was used as a measure of complex stabilities. The standard deviations of experimentally derived CE_50%_ of complexes were below 3% (for majority of experimental points about 1%) for various experimental parameters such as ion-spray voltage (−3.5 kV and −4.5 kV) and MRM scanning time.

#### Nuclear magnetic resonance (NMR)

The ^1^H NMR spectra were recorded on Bruker Avance 400 MHz spectrometer. 2D ROESY NMR experiments were performed on a Varian VNMRS 600 MHz spectrometer with 200 ms mixing time. Spectra of native CDs, PTX-Na_2_, and its complexes were recorded in D_2_O and DMSO-*d*_6_. All 2D NMR measurements were performed in D_2_O. Chemical shifts are reported in ppm relative to DMSO (δ = 2.5 ppm) and HDO (δ = 4.8 ppm). Chemical shifts for PTX [30] and CDs in D_2_O [31], and DMSO [32] are consistent with the literature data.

#### Ultraviolet-visible spectroscopy (UV–vis)

The UV–visible absorption spectra of PTX-Na_2_, and its respective associates with α-CD, β-CD, and γ-CDs were recorded in phosphate buffered saline (PBS) at pH 7.4 using an Evolution 220 UV–vis spectrometer (Thermo Scientific) in the range of 200–450 nm.

**Evaluation of association constants (*K*_as_) of native CDs with PTX. UV–vis titrations:** The change in absorption was measured as a function of CD concentration. In all experiments the concentration of the guest compound was fixed at 10^−4^ mol/dm^3^. All the measurements were done in phosphate buffered saline (PBS, pH 7.4). Obtained data were fitted with the HypSpec program, which enables global fitting of absorption data at all wavelengths.

#### Infrared spectroscopy (FTIR–ATR)

FTIR–ATR measurements were done using a JACSO FT/IR-6200 Spectrometer at 293 K using high resolution Attenuated Total Reflectance (ATR) technique with crystalline ZnSe.

#### Raman spectroscopy

The measurements were performed using the Renishaw in Via Raman system with a spectral resolution of 5 cm^−1^ and with a 300 mW diode laser emitting a 785 nm line, used as an excitation source. The Raman scattering signal was recorded by a 1024 × 256 pixel RenCam CCD detector. Typically, the spectra were acquired for 60 s, with 10% of the laser power.

## Supporting Information

File 1 Copies of MS, UV, NMR, IR and Raman spectra.
