# Supplementary material for: Structural diversity in the host–guest complexes of the antifolate pemetrexed with native cyclodextrins: gas phase, solution and solid state studies
Source: Beilstein J Org Chem. 2017 Oct 25;13:2252–63. doi: 10.3762/bjoc.13.222 (PMC5669224; doi:10.3762/bjoc.13.222)
Supplement: File 1 — Copies of MS, UV, NMR, IR and Raman spectra. [file Beilstein_J_Org_Chem-13-2252-s001.pdf]

Supporting Information

for

Structural diversity in the host–guest complexes of the antifolate pemetrexed with native cyclodextrins: gas phase, solution and solid state studies

Magdalena Ceborska[*1] Magdalena Zimnicka[2] Aneta Aniela Kowalska[1], Kajetan Dąbrowa[2] and Barbara Repeć[2]

Address: [1]Institute of Physical Chemistry, Polish Academy of Sciences, Kasprzaka 44/52, 01-224 and [2]Institute of Organic Chemistry, Polish Academy of Sciences, Kasprzaka 44/52, 01-224 Warsaw, Poland

Email: Magdalena Ceborska - mceborska@ichf.edu.pl

*Corresponding author

Copies of MS, UV, NMR, IR and Raman spectra.

Contents

1. Copies of the mass spectra for α-, β-, and γ-CD complexes with a PTX dianion 2
2. UV–vis 4
3. ^{1}H and 2D NMR spectra in D_2O 6
4. ^{1}H spectra in DMSO-*d*6 13
5. FTIR-ATR and Raman 20

1. Copies of the mass spectra for α–, β–, and γ–CD complexes with a PTX dianion

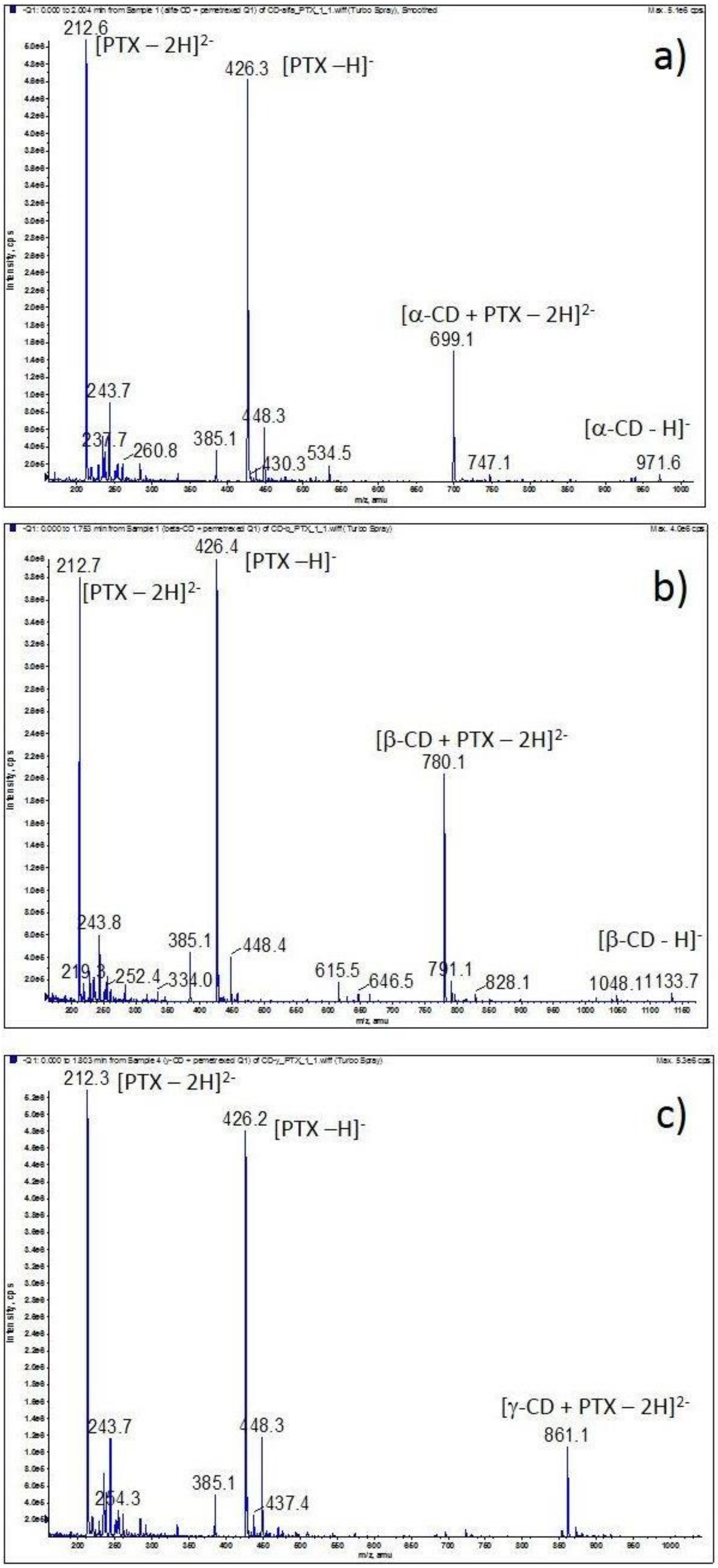

Figure S1. Negative-ESI mass spectra (Q1) of solutions containing: a) α-CD/PTX, b) β-CD/PTX, and c) γ-CD/PTX.

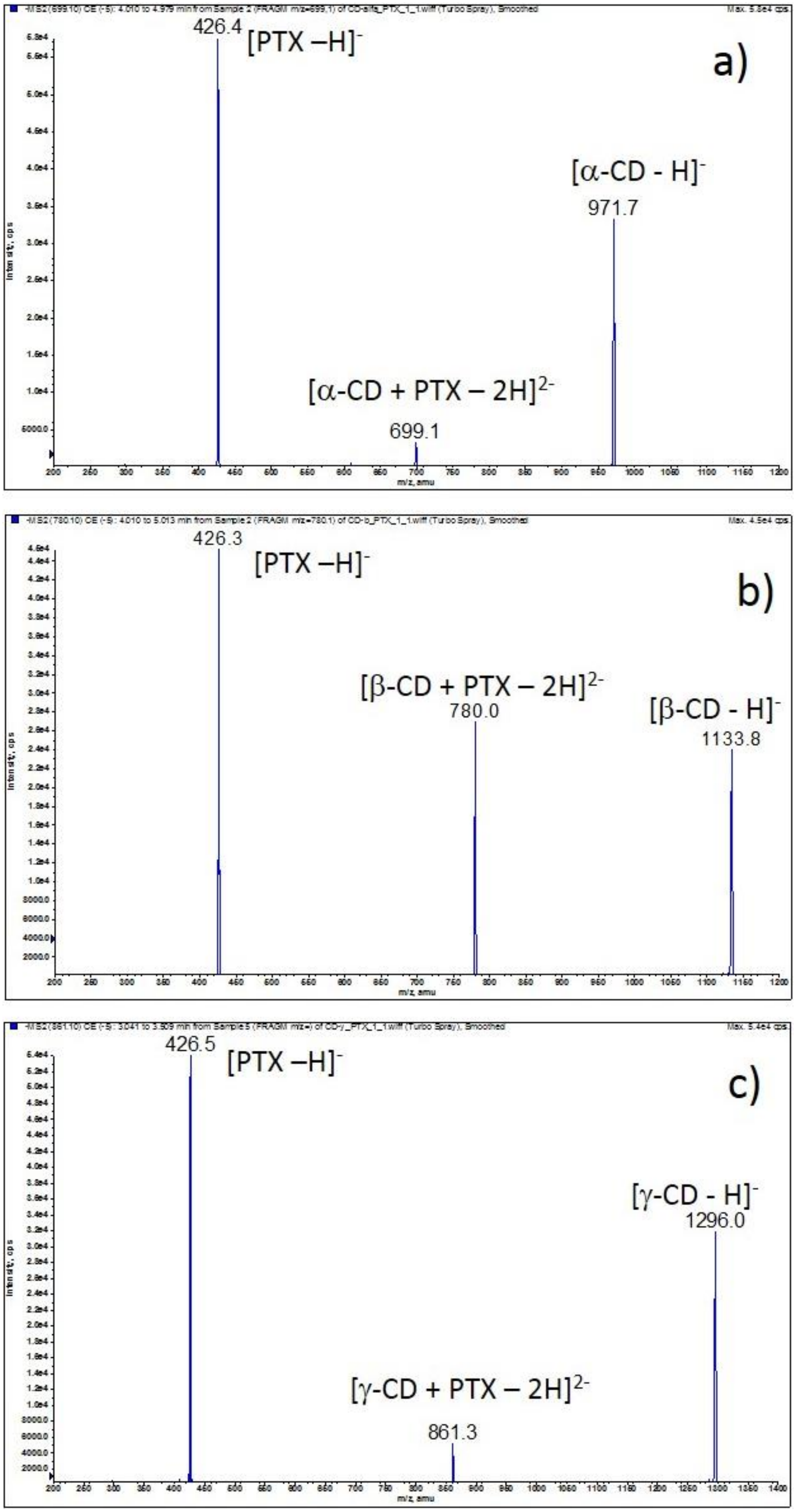

Figure S2. CID spectra of gas-phase complexes of a) α-CD/PTX, b) β-CD/PTX, and c) γ-CD/PTX.

2. Ultra-Visible Spectroscopy (UV-VIS)

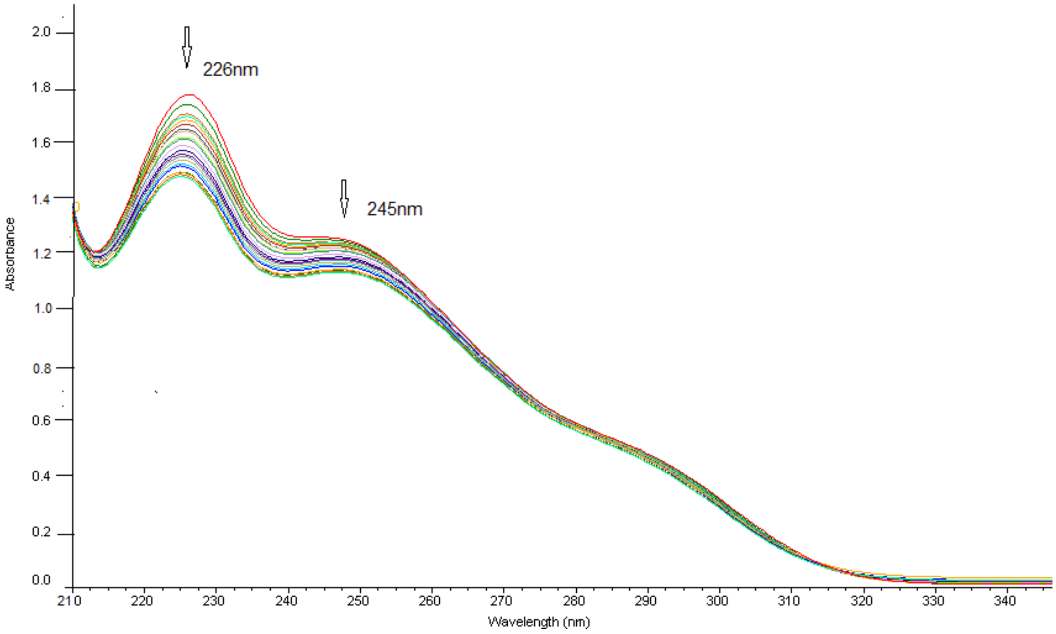

Figure S3. Absorption spectra of PTX upon titration with β-CD.

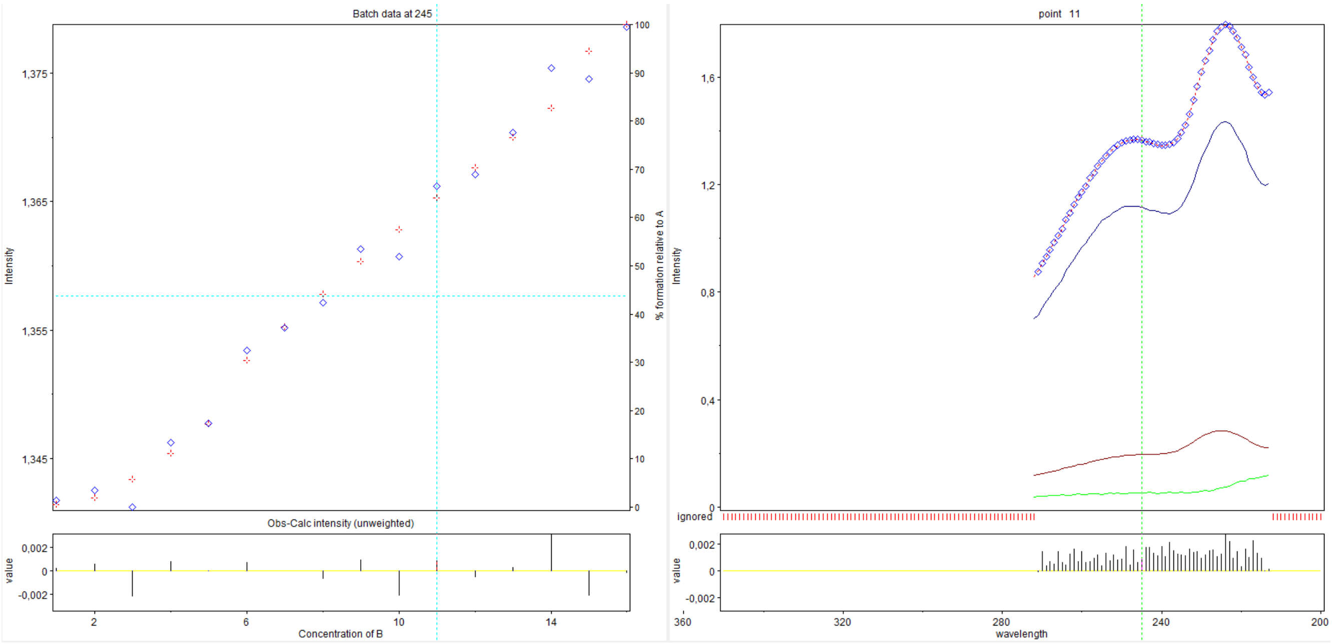

Figure S4. Titration plot for titration of PTX with β-CD (shown at λ = 245 (left) and for 11th titration point (right).

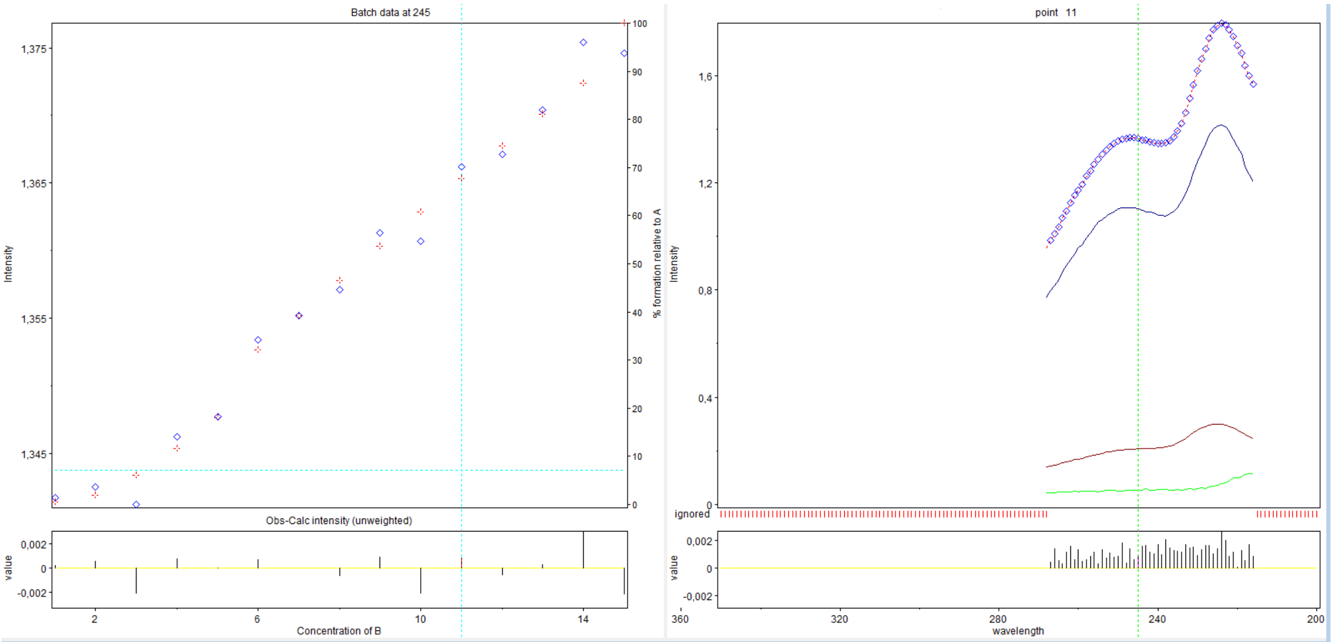

Figure S5. Titration plot for titration of PTX with γ-CD (shown at λ = 245 (left) and for the 11th titration point (right).

3. ^{1}H and 2D NMR spectra in D_2O

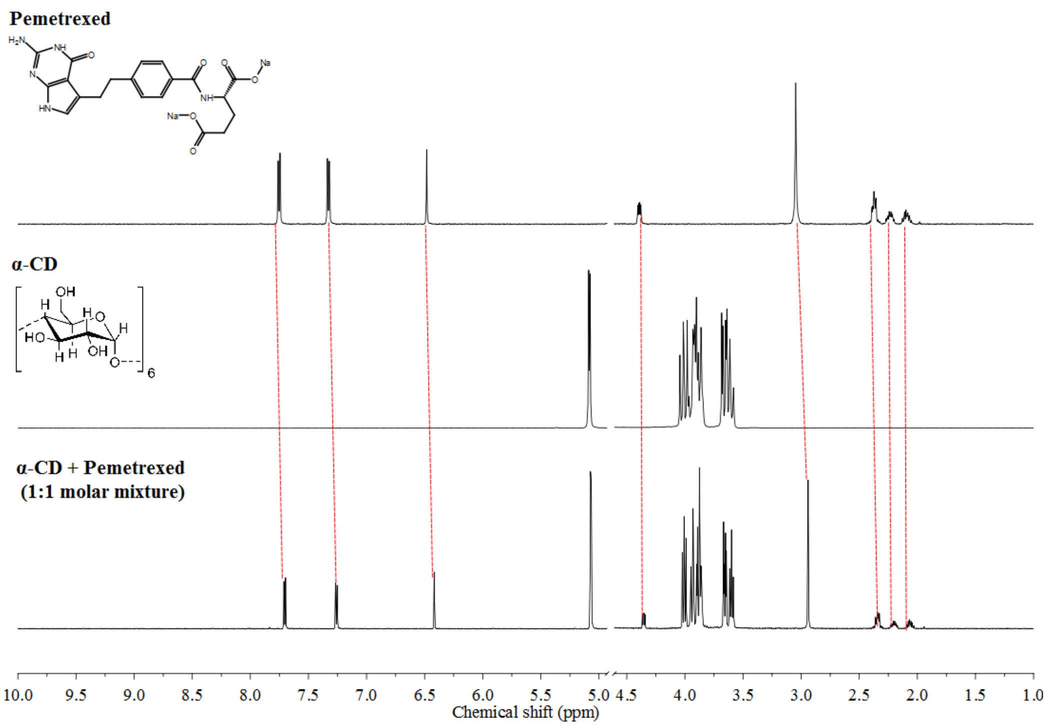

Figure S6. ^{1}H NMR stacked spectra of PTX, α-CD, and their 1:1 molar mixture (from top-to-bottom) in D_2O at 298 K.

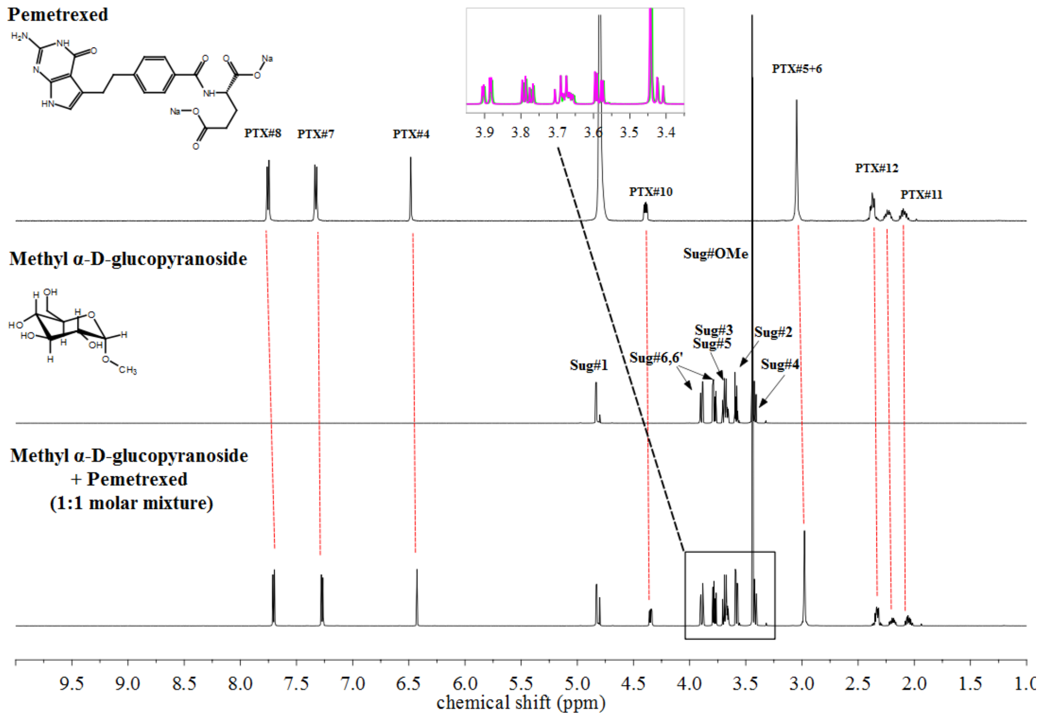

Figure S7. ^{1}H NMR stacked spectra of PTX, methyl α-D-glucopyranoside, and their 1:1 mixture (from top-to-bottom) in D_2O at 298 K; inset - enlargement of the spectrum exemplifying marginal chemical shift changes of the sugar' protons.

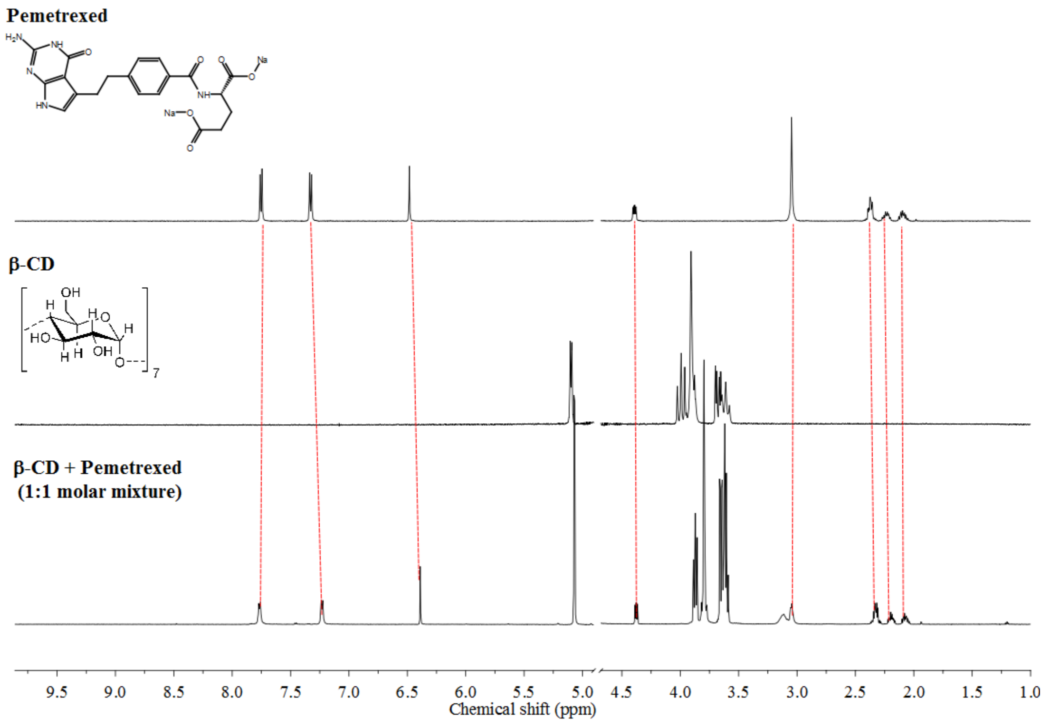

Figure S8. ^{1}H NMR stacked spectra of PTX, β-CD, and their 1:1 molar mixture (from top-to-bottom) in D_2O at 298 K.

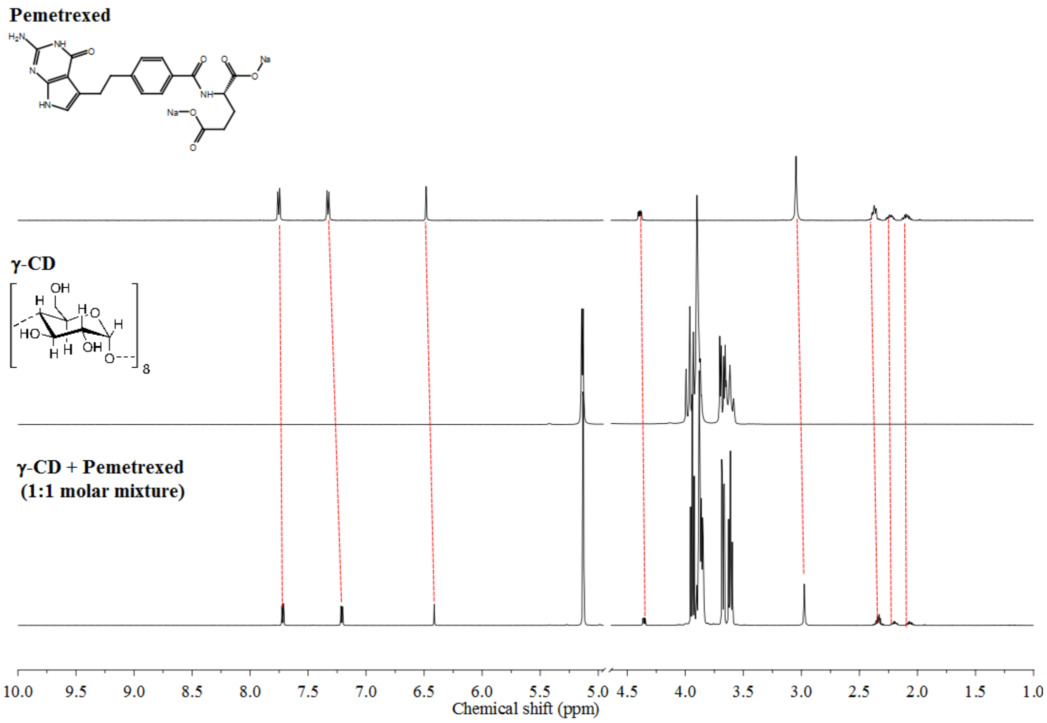

Figure S9. ^{1}H NMR stacked spectra of PTX, γ-CD, and their 1:1 molar mixture (from top-to-bottom) in D_2O at 298 K.

a)

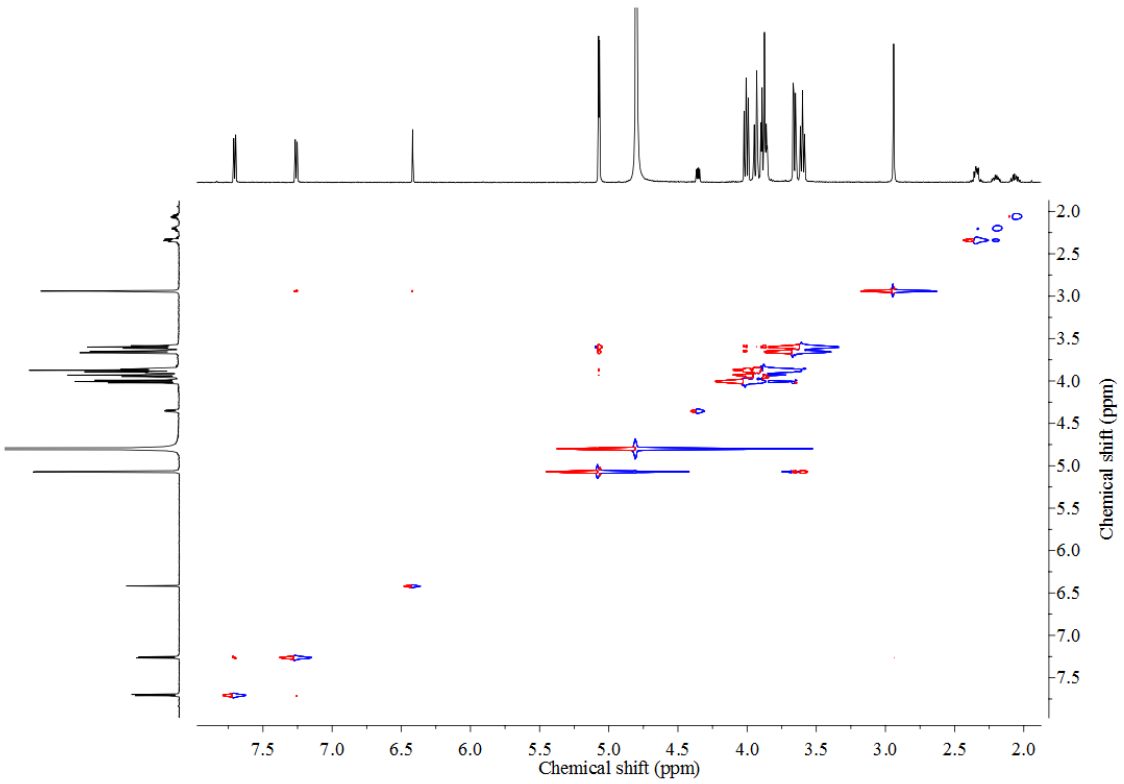

b)

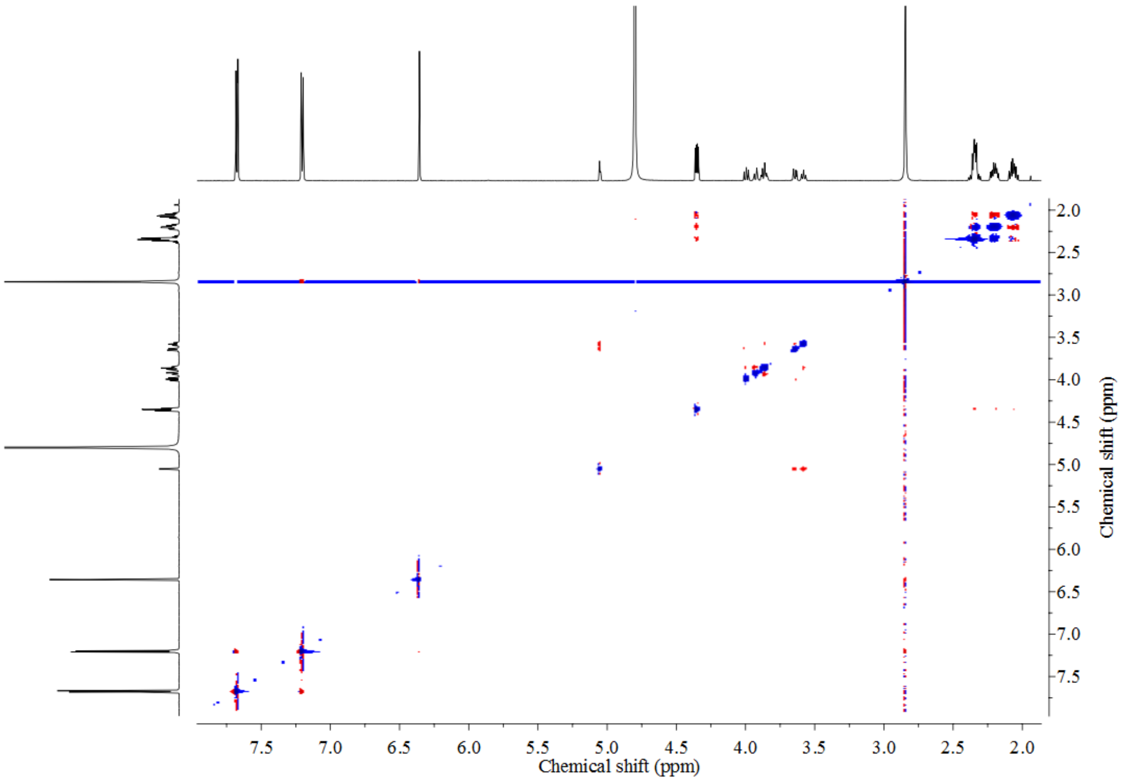

Figure S10. 2D ROESY NMR spectra of a 1:1 (a) and 1:16 (b) molar mixture of α-CD and PTX in D_2O at 298 K.

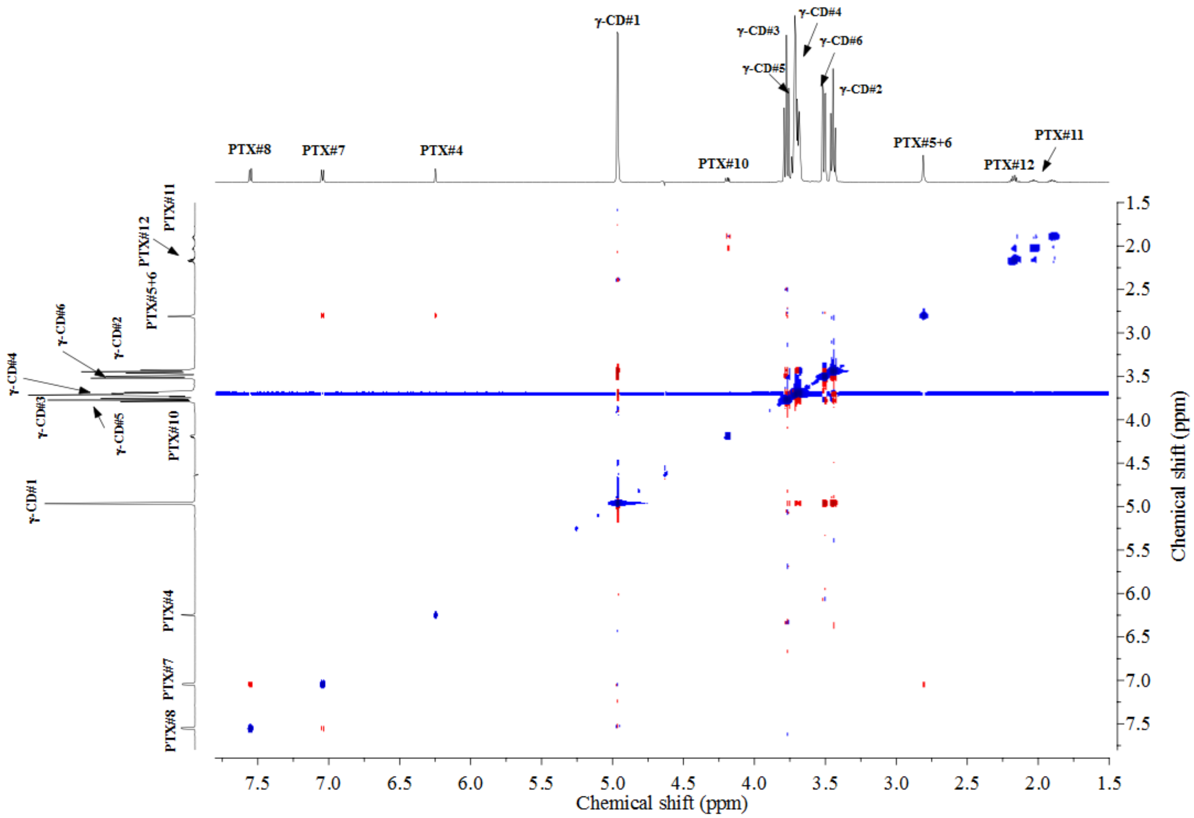

Figure S11. 2D ROESY NMR spectra of a 1:1 molar mixture of γ-CD and PTX in D_2O at 298 K.

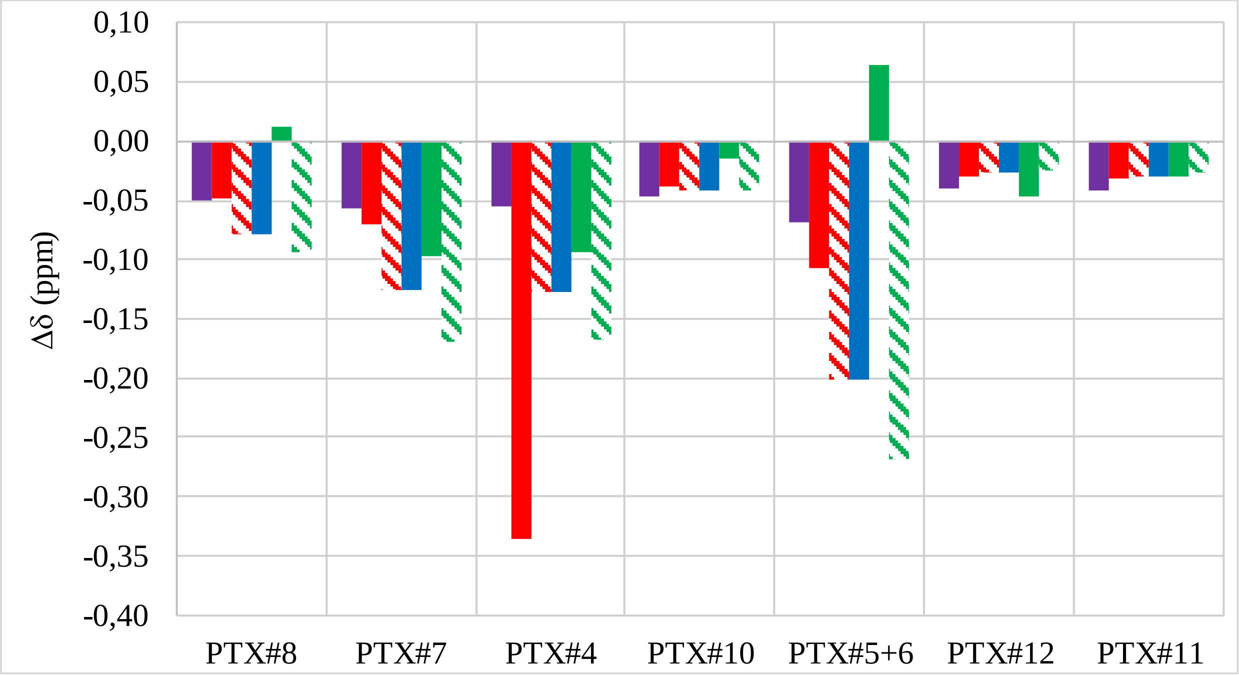

Figure S12. Experimental chemical shift changes (Δδ) of the PTX guest in the 1:1 (solid) and 1:16 (dashed) molar mixtures with an appropriate sugar in D_2O (methyl α-D-glucopyranoside – violet, α-CD/PTX – red, β-CD/PTX – blue, γ-CD/PTX – green).

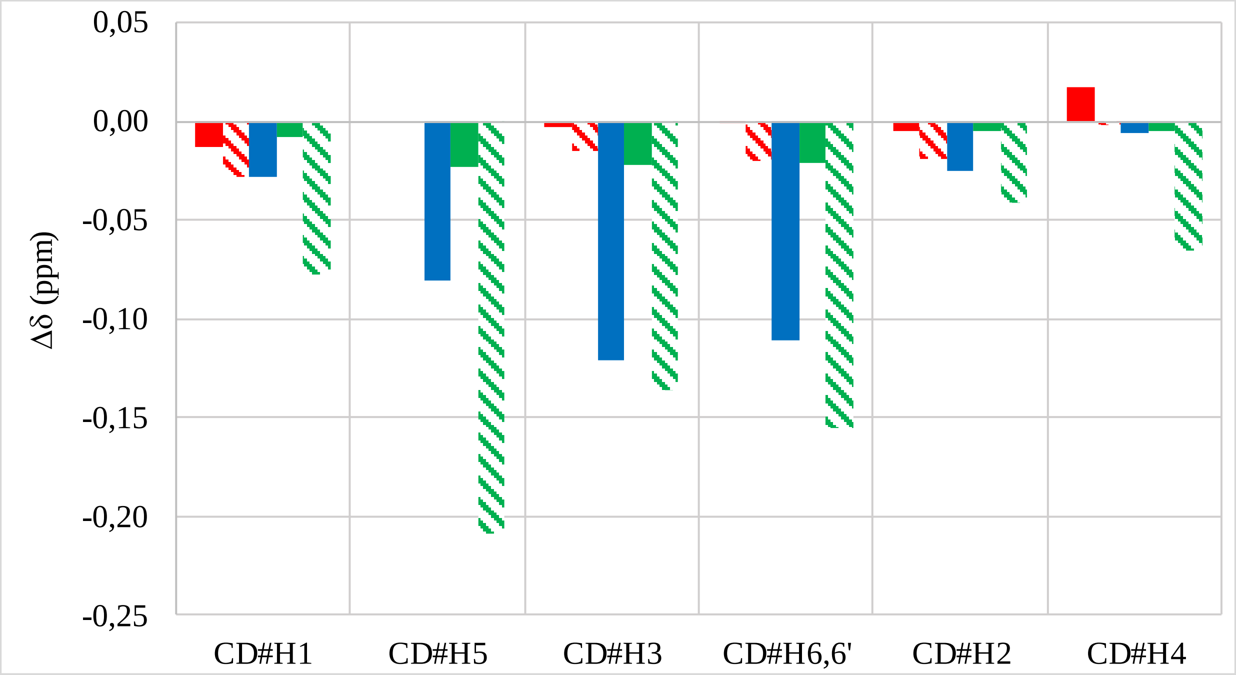

Figure S13. Experimental chemical shift changes (Δδ) of the CD host in the presence of equimolar (solid) and a 16-fold excess (dashed) of PTX guest in D_2O (α-CD – red, β-CD – blue, γ-CD – green).

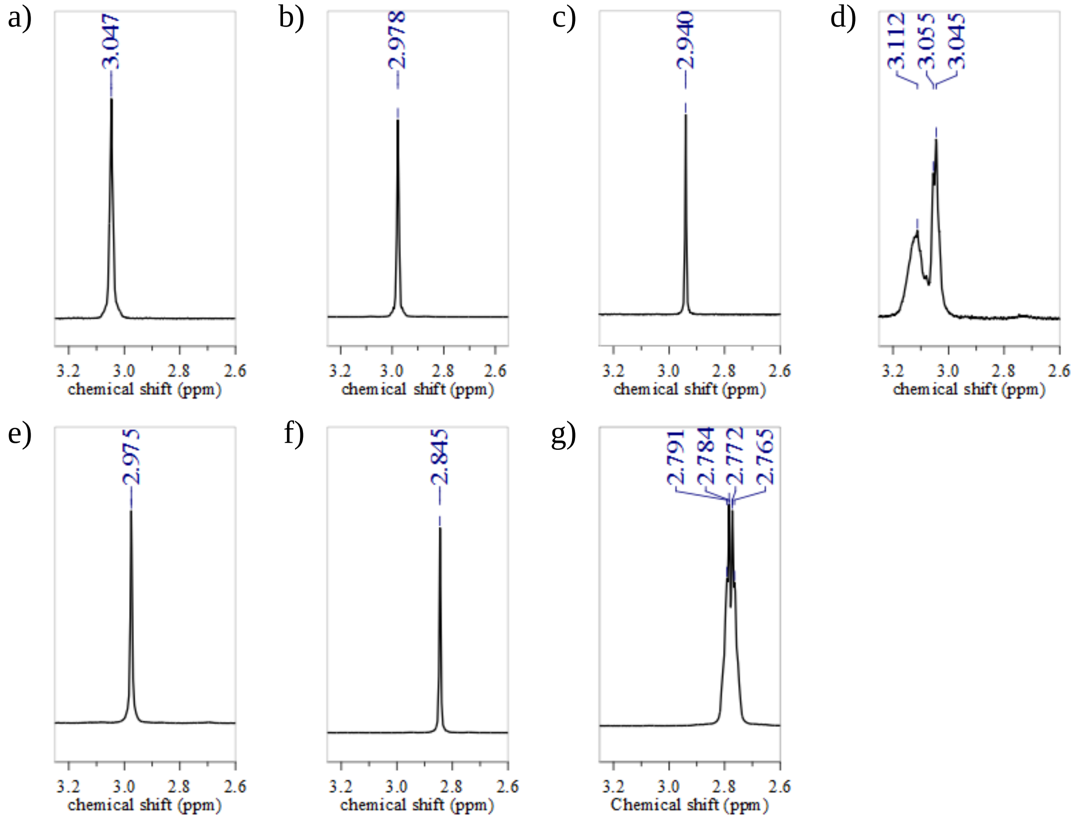

Figure S14. Partial ^{1}H NMR spectra showing signals of the bridging ethylene moiety (PTX#H5+H6) of the PTX guest: (a) free PTX, equimolar mixtures with (b) methyl α-D-glucopyranoside, (c) α-CD, (d) β-CD, (e) γ-CD, and 1:16 molar mixtures (sugar/PTX) with (f) α-CD and (g) γ-CD.

Table S1. Chemical shifts (δ) for protons of α-CD, PTX, and their 1:1 mixture (in D_2O) [a]

Signal	δ_{free}	$\delta_{complex}$	$\Delta\delta_{complex}$
PTX#8	7.75	7.70	-0.05
PTX#7	7.33	7.26	-0.07
PTX#4	6.48	6.15	-0.34
PTX#10	4.39	4.35	-0.04
PTX#5+6	3.05	2.94	-0.11
PTX#12	2.37	2.34	-0.03
PTX#11	2.16	2.13	-0.03
α−CD#H1	5.08	5.07	-0.01
α−CD#H5	3.90	n.r.	n.r.
α−CD#H3	4.01	4.01	0.00
α−CD#H6.6'	3.86	3.86	0.00
α−CD#H2	3.66	3.66	-0.01
α−CD#H4	3.58	3.60	0.02

[a] in D_2O at 298 K; δ_{free}, $\Delta\delta_{complex}$ (defined as $\delta_{complex} - \delta_{free}$) are given in ppm; only protons for which important changes appear are presented; n.r. = not resolved due to overlapping with other signals.

Table S2. Chemical shifts for protons of methyl α−D−glucopyranoside, PTX, and their 1:1 mixture (in D_2O) [a]

Signal	δ_{free}	$\delta_{complex}$	$\Delta\delta_{complex}$
PTX#8	7.75	7.70	-0.05
PTX#7	7.33	7.27	-0.06
PTX#4	6.48	6.43	-0.05
PTX#10	4.39	4.35	-0.05
PTX#5+6	3.05	2.98	-0.07
PTX#12	2.37	2.33	-0.04
PTX#11	2.16	2.12	-0.04
Sug#H1	4.83	4.83	0.00
Sug#H5	3.65	3.66	0.00
Sug#H3	3.69	3.69	0.00
Sug#H6,6'	3.89, 3.78	3.89, 3.78	0.00, 0.00
Sug#H2	3.58	3.58	0.00
Sug#H4	3.42	3.42	0.00
Sug#OMe	3.44	3.44	0.00

[a] in D_2O at 298K; δ_{free}, $\Delta\delta_{complex}$ (defined as $\delta_{complex} - \delta_{free}$) are given in ppm; only protons for which important changes appear are presented.

Table S3. Chemical shifts (δ) for protons of β−CD, PTX, and their 1:1 mixture (in D_2O) [a]

Signal	δ_{free}	$\delta_{complex}$	$\Delta\delta_{complex}$
PTX#8	7.75	7.77	0.01
PTX#7	7.33	7.23	-0.10
PTX#4	6.48	6.39	-0.09
PTX#10	4.39	4.38	-0.01
PTX#5+6	3.05	3.11	0.06
PTX#12	2.37	2.33	-0.05
PTX#11	2.16	2.13	-0.03
β−CD#H1	5.10	5.07	-0.03
β−CD#H5	3.88	3.80	-0.08
β−CD#H3	3.99	3.87	-0.12
β−CD#H6,6'	3.91	3.80	-0.11
β−CD#H2	3.68	3.65	-0.02
β−CD#H4	3.61	3.61	-0.01

[a] in D_2O at 298K; δ_{free}, $\Delta\delta_{complex}$ (defined as $\delta_{complex} - \delta_{free}$) are given in ppm; only protons for which important changes appear are presented.

Table S4. Chemical shifts (δ) for protons of γ–CD, PTX, and their 1:1 mixture (in D_2O) [a]

Signal	δ_{free}	$\delta_{complex}$	$\Delta\delta_{complex}$
PTX#8	7.75	7.72	-0.04
PTX#7	7.33	7.21	-0.12
PTX#4	6.48	6.41	-0.07
PTX#10	4.39	4.36	-0.04
PTX#5+6	3.05	2.98	-0.07
PTX#12	2.37	2.34	-0.03
PTX#11	2.16	2.13	-0.03
γ−CD#H1	5.14	5.13	-0.01
γ−CD#H5	3.87	3.85	-0.02
γ−CD#H3	3.96	3.94	-0.02
γ−CD#H6,6'	3.90	3.88	-0.02
γ−CD#H2	3.68	3.68	0.00
γ−CD#H4	3.62	3.61	0.00

[a] in D_2O at 298K; δ_{free}, $\Delta\delta_{complex}$ (defined as $\delta_{complex} - \delta_{free}$) are given in ppm; only protons for which important changes appear are presented.

4. ^{1}H spectra in DMSO-d_6

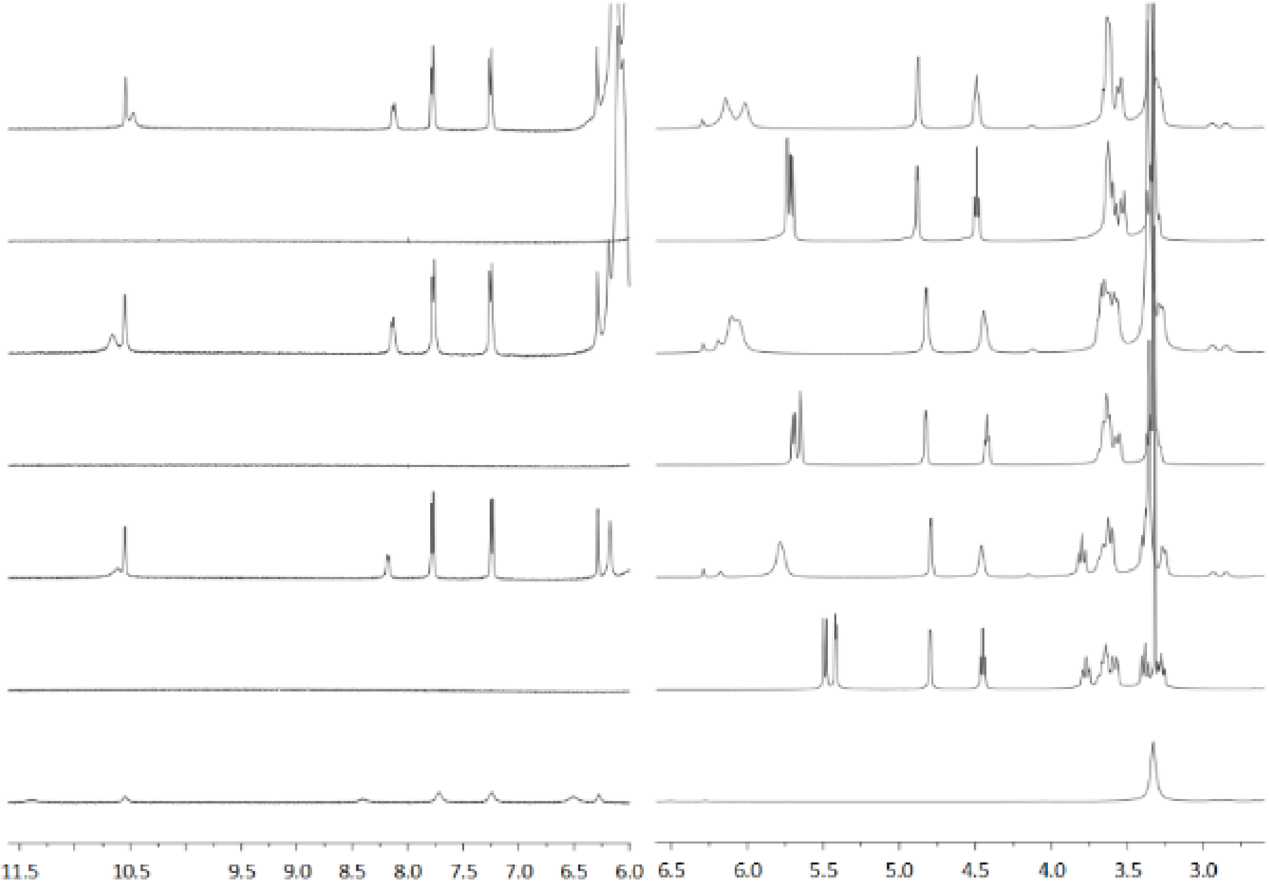

Figure S15. Stacked spectra of the free PTX, CDs, and complexes of CDs with PTX in DMSO-d_6 (from bottom to top: PTX, α-CD, α-CD+PTX, β-CD, β-CD+PTX, γ-CD, γ-CD+PTX). Notice that broad signals belonging to the PTX molecule became sharp after addition of CDs. This clearly indicates complex formation which increases solubility of PTX salt in that particular solvent.

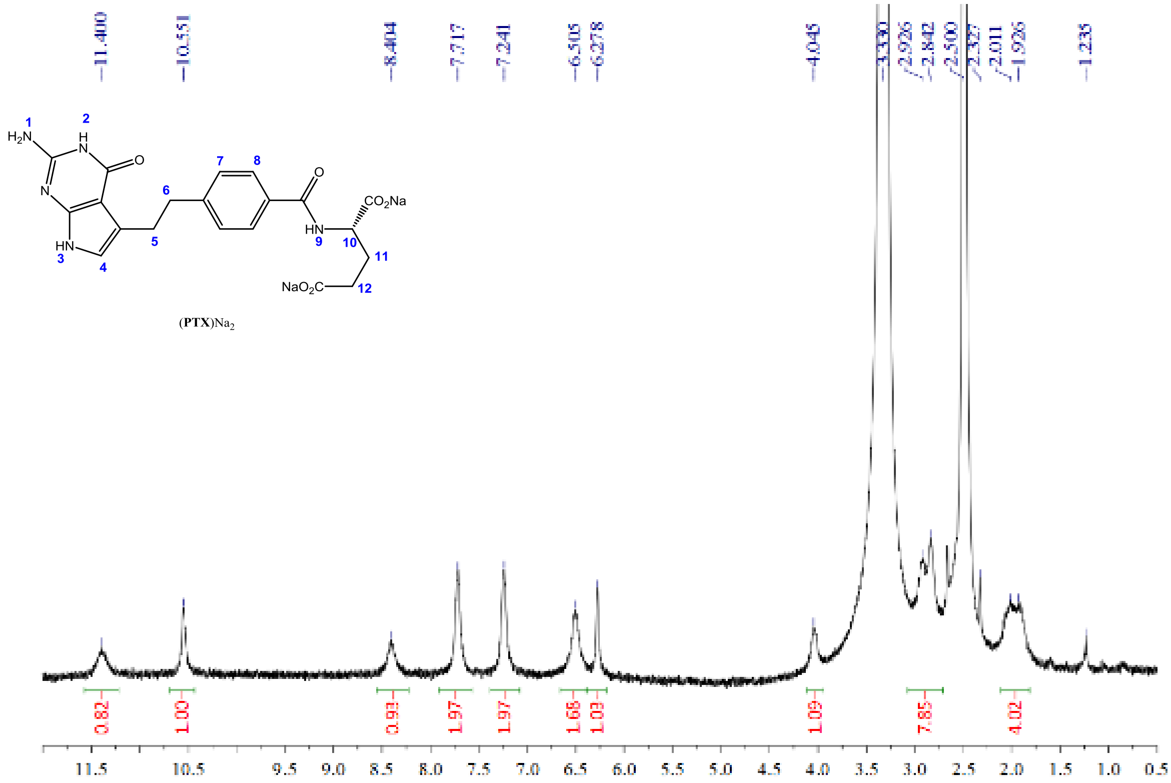

Figure S16. ^{1}H NMR (400 MHz) spectrum of disodium salt of PTX in DMSO-d_6.

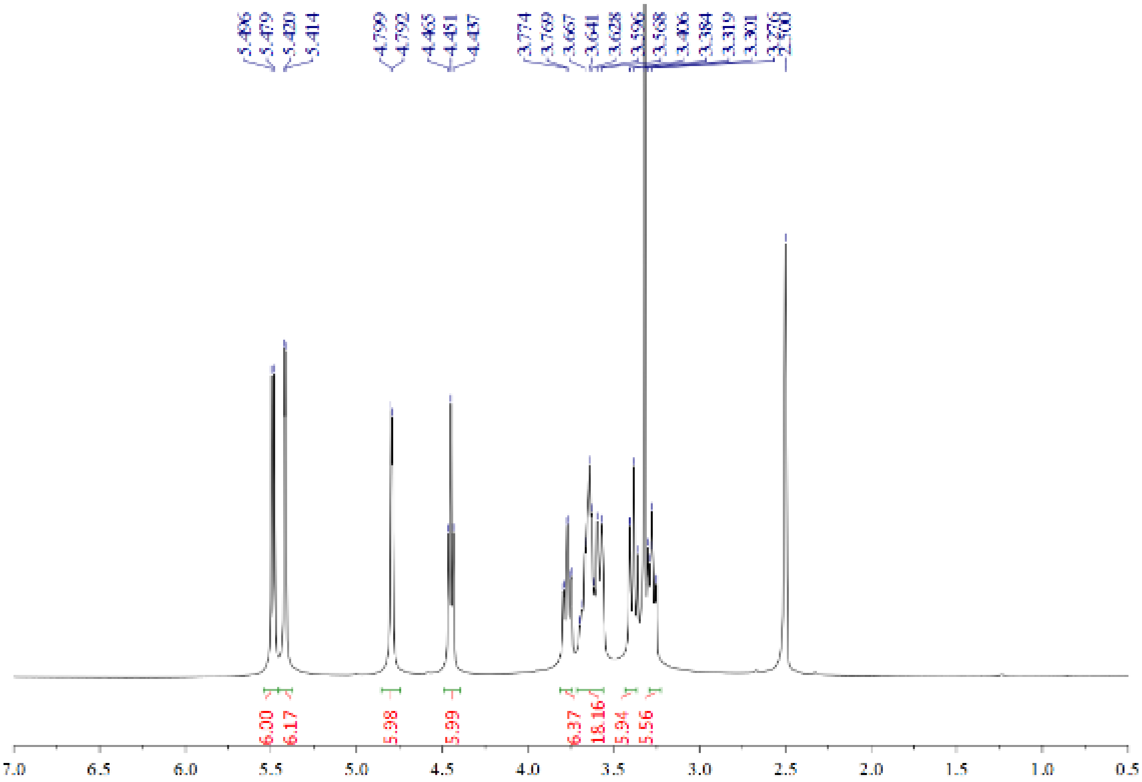

Figure S17. ^{1}H NMR (400 MHz) spectrum of free α-CD in DMSO-d_6.

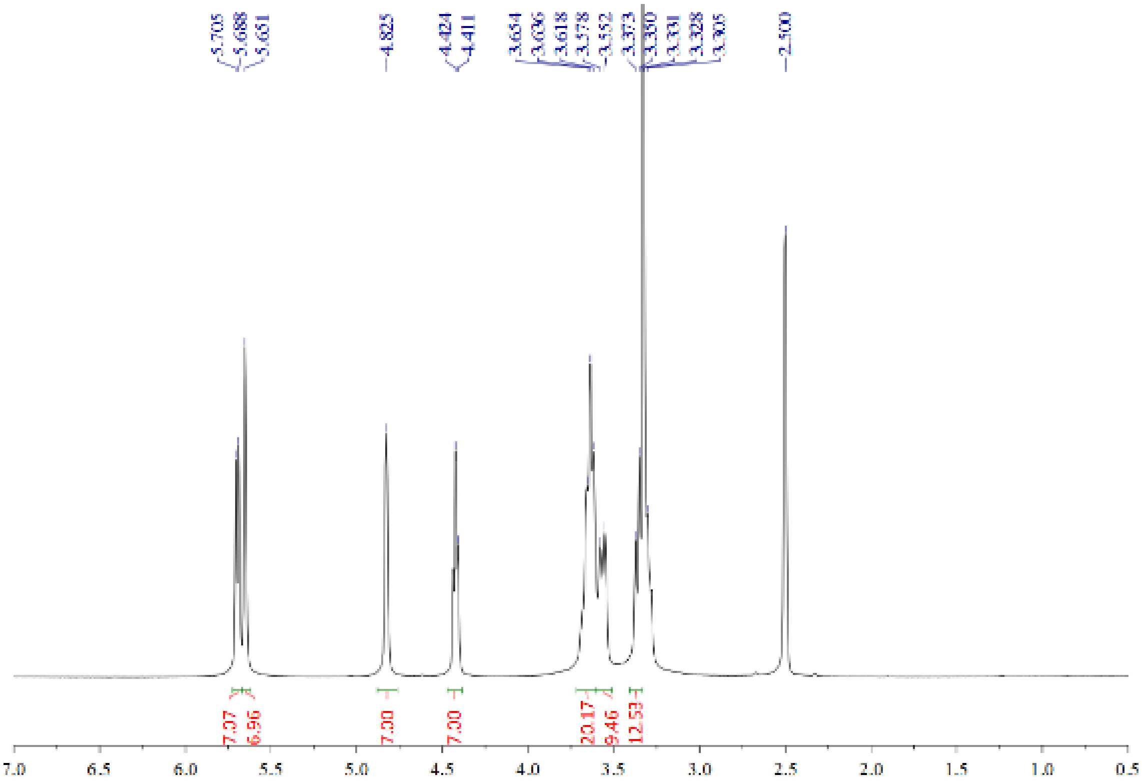

Figure S18. ^{1}H NMR (400 MHz) spectrum of free β-CD in DMSO-d_6.

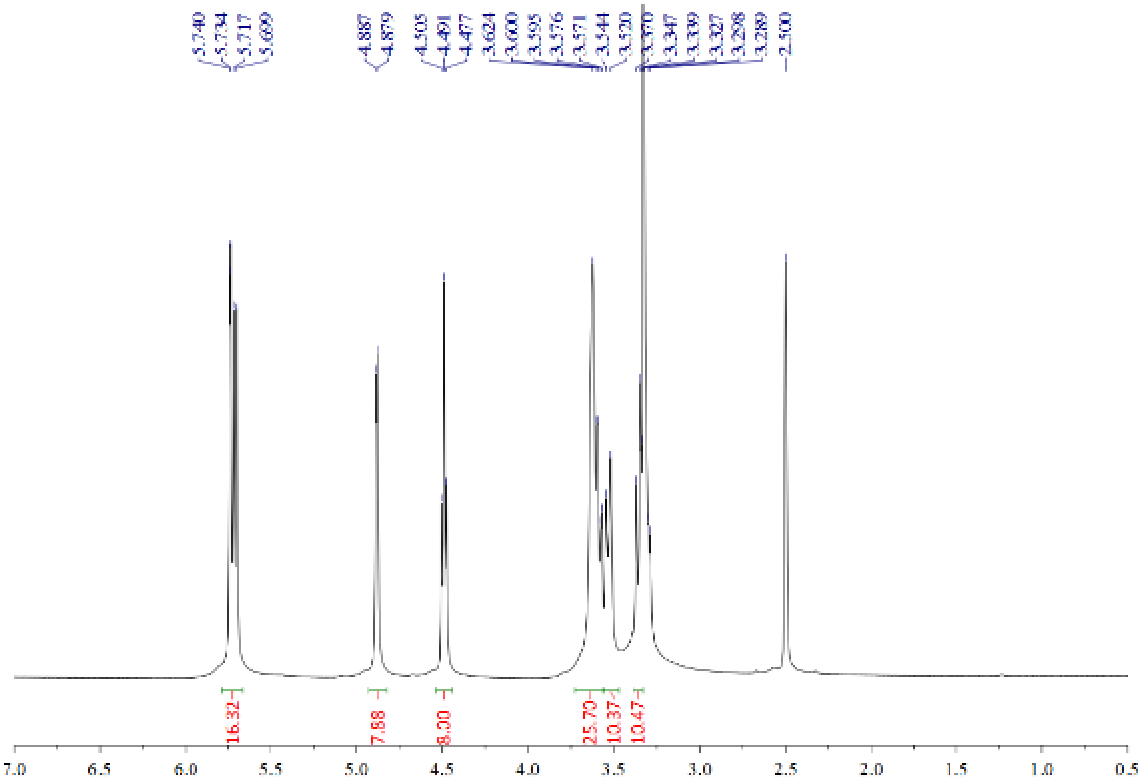

Figure S19. ^{1}H NMR (400 MHz) spectrum of free γ-CD in DMSO-d_6.

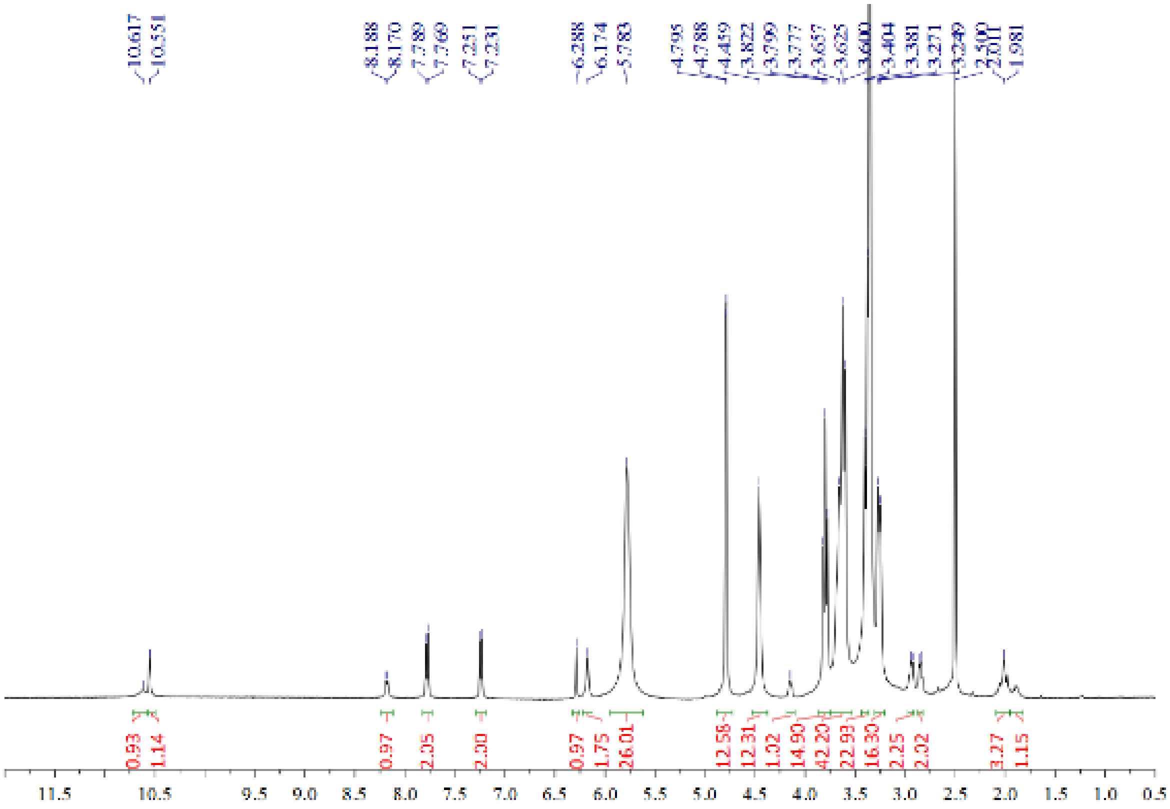

Figure S20. ^{1}H NMR (400 MHz) spectrum of a mixture of α-CD and PTX (1:1 molar ratio) in DMSO-d_6.

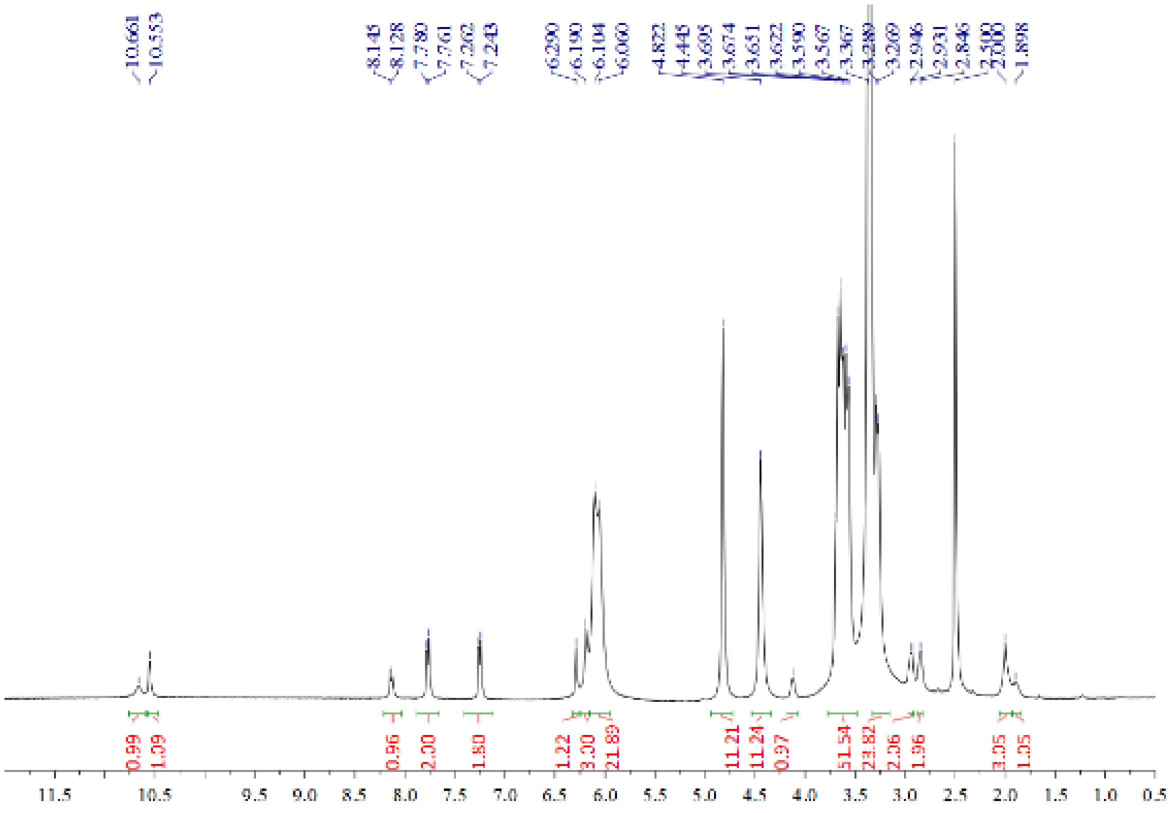

Figure S21. ^{1}H NMR (400 MHz) spectrum of a mixture of β-CD and PTX (1:1 molar ratio) in DMSO-d_6.

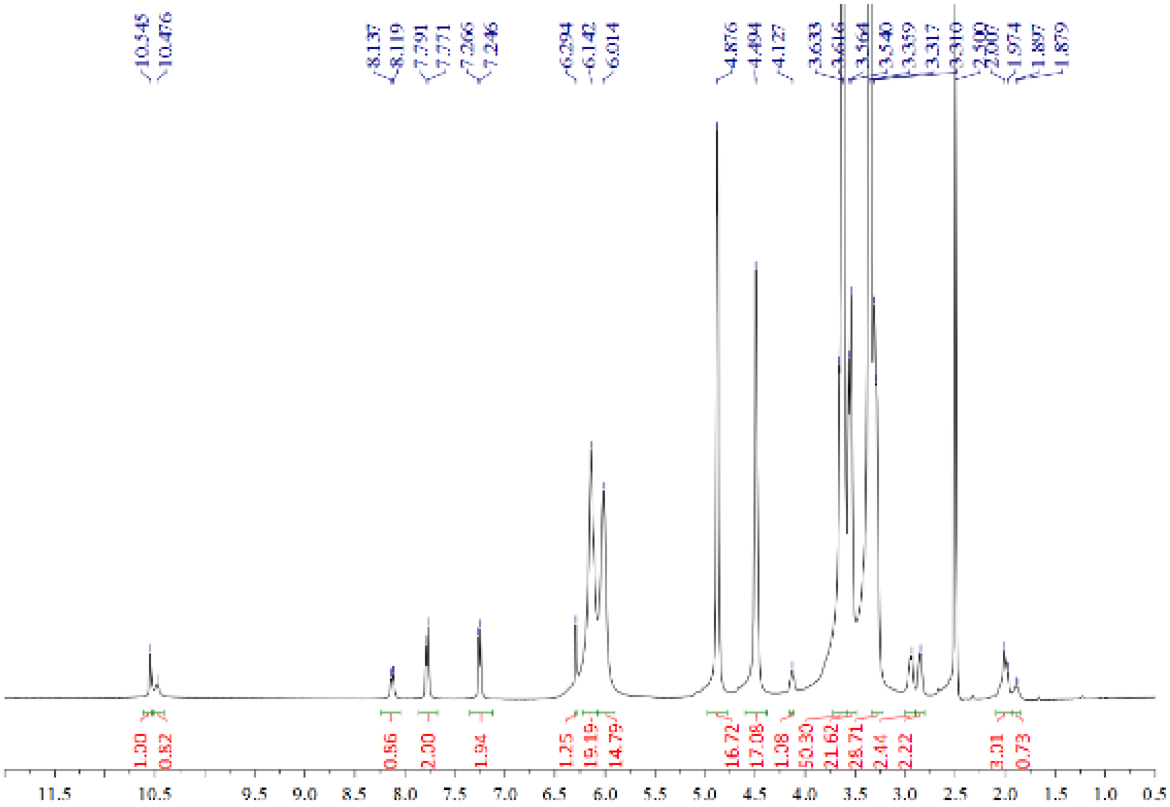

Figure S22. ^{1}H NMR (400 MHz) spectrum of a mixture of γ-CD and PTX (1:1 molar ratio) in DMSO-d_6.

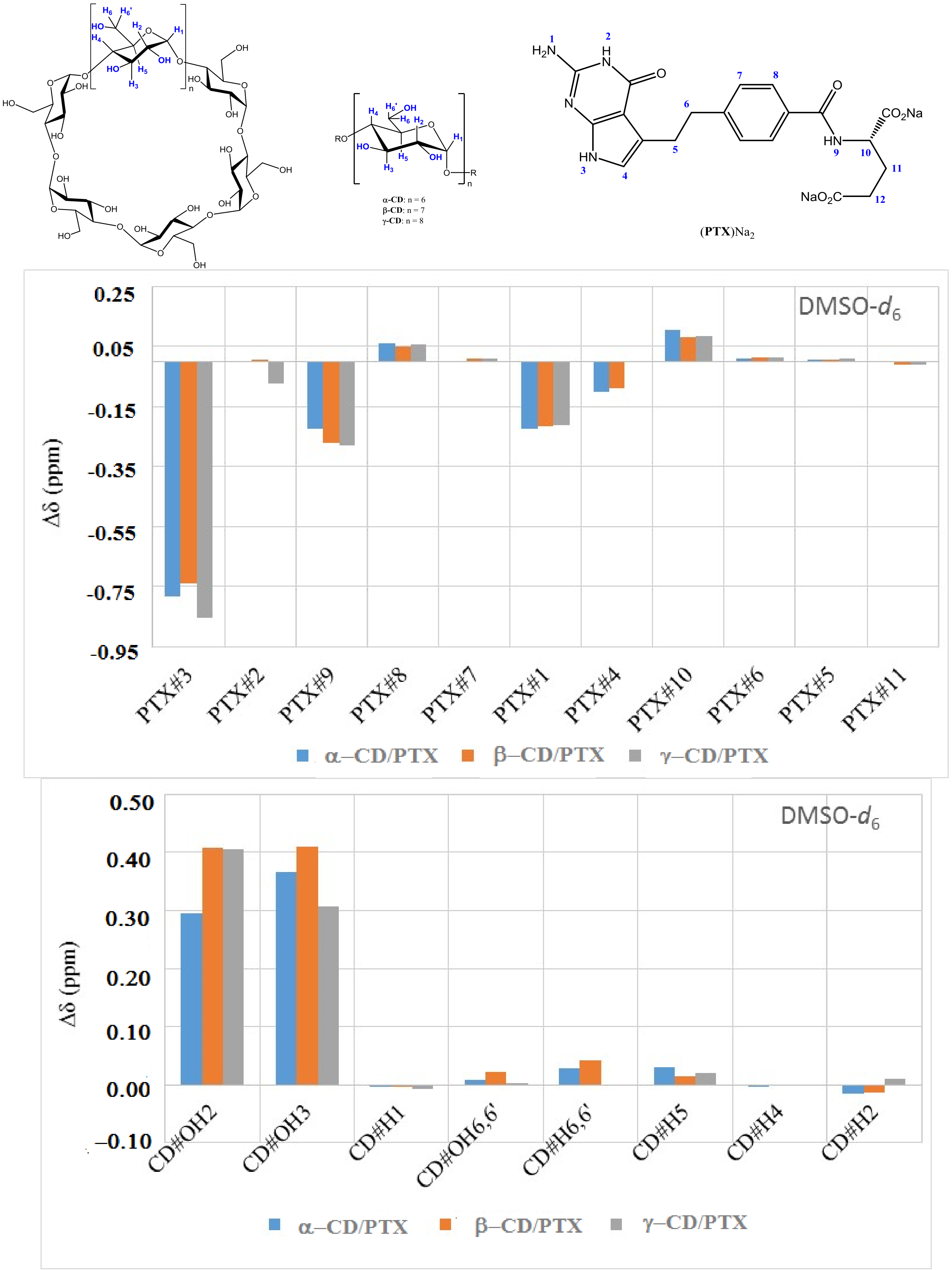

Figure S23. Experimental changes in chemical shifts of PTX (top) and CD (bottom) upon 1:1 host:guest complex formation in DMSO-d_6.

Table S5. Changes in chemical shifts of PTX (added as disodium salt) upon 1:1 complex formation with CD in DMSO-d_6 [a]

Signal	PTX$_{free}$	α–CD/PTX		β–CD/PTX		γ–CD/PTX	
	δ_F	δ_C	Δδ	δ_C	Δδ	δ_C	Δδ
PTX#3	11.400	10.617	-0.783	10.661	-0.739	10.545	-0.855
PTX#2	10.551	10.551	0.000	10.553	0.002	10.476	-0.075
PTX#9	8.404	8.179	-0.225	8.134	-0.270	8.125	-0.279
PTX#8	7.717	7.777	0.060	7.766	0.049	7.775	0.058
PTX#7	7.241	7.240	-0.001	7.250	0.009	7.252	0.011
PTX#1	6.505	6.280	-0.225	6.290	-0.215	6.291	-0.214
PTX#4	6.278	6.175	-0.103	6.190	-0.088	— [b]	— [b]
PTX#10	4.045	4.150	0.105	4.124	0.079	4.128	0.083
PTX#6	2,926	2.936	0.010	2.939	0.013	2.941	0.015
PTX#5	2.842	2.849	0.007	2.848	0.006	2.851	0.009
PTX#11	2.011	2.011	0.000	2.000	-0.011	2.002	-0.009

[a] δ_F, δ_C, and Δδ are given in ppm; δ_F and δ_C indicate chemical shift of the uncomplexed and complexed form, respectively; Δδ = δ_C - δ_F; [b] signal not visible.

Table S6. Changes in chemical shifts of CD upon 1:1 complex formation with PTX in DMSO-d_6 [a]

Signal	α–CD$_{free}$	α–CD/PTX		β–CD$_{free}$	β–CD/PTX		γ–CD$_{free}$	γ–CD/PTX	
	δ_F	δ_C	Δδ	δ_F	δ_C	Δδ	δ_F	δ_C	Δδ
CD#OH2	5.488	5.783	0.296	5.696	6.104	0.408	5.737	6.142	0.405
CD#OH3	5.417	5.783	0.366	5.651	6.060	0.409	5.708	6.014	0.306
CD#H1	4.796	4.792	-0.004	4.827	4.823	-0.004	4.883	4.876	-0.007
CD#OH6,6'	4.451	4.459	0.008	4.423	4.445	0.022	4.491	4.494	0.003
CD#H6,6'	3.772	3.799	0.027	3.633	3.674	0.041	3.624	3.624	0.000
CD#H5	3.582	3.613	0.031	3.565	3.579	0.014	3.532	3.552	0.020
CD#H4	3.384	3.381	-0.003	3.350	— [b]	— [b]	3.347	— [b]	— [b]
CD#H2	3.276	3.260	-0.016	3.293	3.279	-0.014	3.289	3.299	0.009

[a] δ_F, δ_C, and Δδ are given in ppm; δ_F and δ_C indicate chemical shift of the uncomplexed and complexed form, respectively; Δδ = δ_C - δ_F; [b] signal not visible.

5. FTIR-ATR and Raman

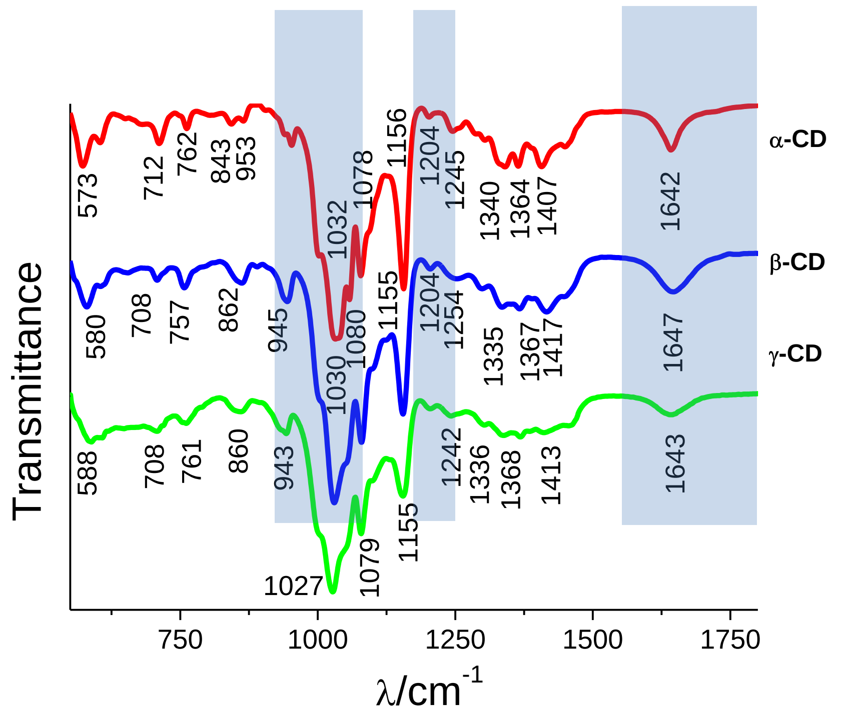

Figure S24. The FTIR spectra of native CDs.

Table S7. FTIR band position assignments for native cyclodextrins.

FTIR band positions [cm^{-1}]			Assignments
α-CD	β-CD	γ-CD	
762	758	761	$(COO)^{-}$
843	862	860	Skeletal "C–C" stretching, CH out of plane bending (aromatic and skeletal)
953	945	943	"C=O" stretching, in plane bending of CCH
1032	1030	1027	C-O in C-O-C stretching
1078	1080	1079	"C–O", "C–C" stretching, in plane bending of OCH
1156		1155	In plane bending CCH (aromatic and skeletal), "C–O" symmetric stretching
1340	1335	1336	"C–O–C" stretching, out of plane bending of CH_3, in plane bending of aromatic CCH
1407	1417	1413	OCH, CCH
1450		1457	In plane bending of CH_3
1642	1647	1643	Bending vibration of the "O–H", "C=C" stretching

Table S8. FTIR band position assignments for PTX and its complexes with α-, β-, and γ-CDs.

FTIR band positions [cm^{-1}]				Assignments
PTX	α-CD /PTX	β-CD/ PTX	γ-CD/ PTX	
676				$(COO)^-$
784		757, 786	759	Skeletal "C–C" stretching, CH out of plane bending (aromatic and skeletal)
812<br>833<br>853	869	862	860	CH_3 and CH out of plane bending of aromatic CCH and skeletal CCH
906<br>952	952	945	943	"C=O" stretching, in plane bending of CCH
	1028	1030	1027	"C–O–C" stretching, out of plane bending of CH_3, in plane bending of aromatic CCH
1083, 1096	1077	1080	1079	"C–O", "C–C" stretching, in plane bending of OCH
1155	1152	1155	1155	CH overtone stretching, "C–C" stretching
1272 1287				CH in plane bending of "C=CH", aromatic "C–O" stretching
1320, 1355, 1375				In plane bending of CH, enolic COH, skeletal CCC
1404	1414	1417	1413	OCH, CCH
1445, 1507, 1531<br>1569, 1603	1502<br>1609	1500<br>1602	1496<br>1528	"C=C" stretching, in plane bending of CH_3, "C=O" stretching, "C–C=O" in plane bending,
1623, 1671	1638	1647	1632, 1663	"C=O", ring "C=C" stretching

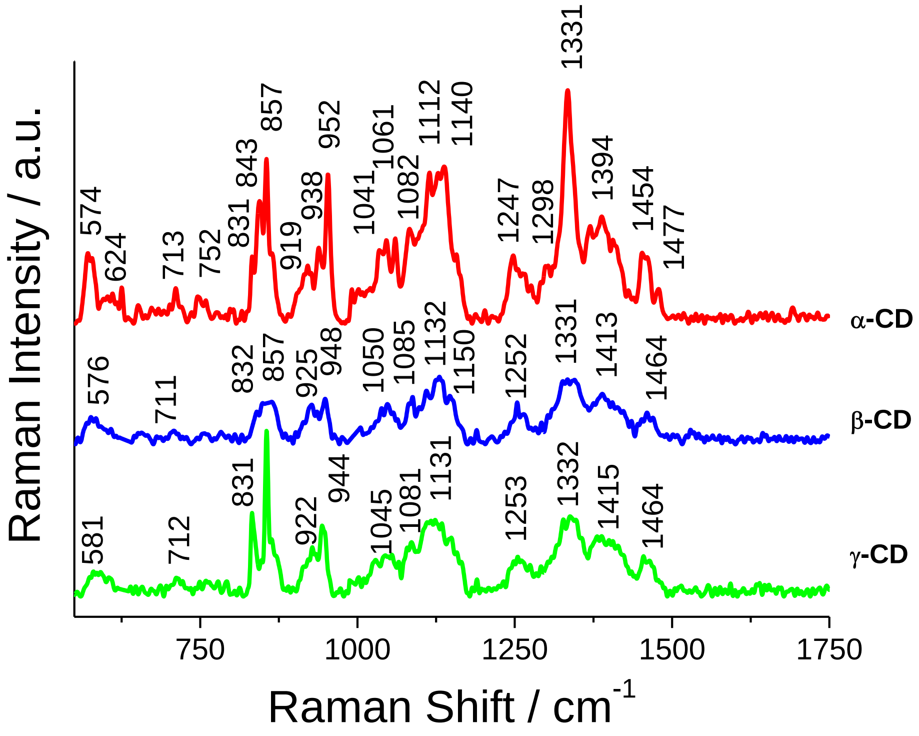

Figure S25. The Raman spectra of native CDs.

Table S9. Raman band position assignments for native cyclodextrins.

Raman band positions [cm^{-1}]			Assignments
α-CD	β-CD	γ-CD	
713, 752	711, 755		(COO^-)
831, 843, 857	832, 857	831, 855	(COO^-), (CH3)
919, 938, 952	925, 948	922, 944	Stretching (CC)
1041	1050		Stretching (CN)
1061, 1082	1085	1081	Stretching (CN)
1331	1325, 1331	1332	Wagging of CH_2, CH, OH
1454, 1477	1413, 1464	1415, 1464	In plane bending of CH_3, OH and C=C stretching

Table S10. Raman band position assignments for PTX and its complexes with α–, β–, and γ–CDs.

Raman band positions [cm^{-1}]				Assigments
PTX	α-CD	β-CD	γ-CD	
810	810	769		(COO^-)
834, 857	834, 857	834, 857	831, 855	(COO^-), CH_3
878	887			CH_3
908	923	931, 950		Stretching (CC)
992	992	992	990	Rocking (NH_2) out of plane, ring deformation[(a)]
1038				Stretching (CN), Rocking (OH)(CH)(NH_2) [(a)]
1091	1078	1087	1080	Stretching (CN), Rocking (OH) [(a)]
1159			1150	C=C–H and =C–C=, Rocking (CH)(OH), wagging (NH_2)[(a)]
1195			1190	Rocking (NH_3^+), Rocking (CH)(OH), ring deformations[(a)]
1247, 1273, 1295			1253	Stretching (C–N), bending (N–H), stretching (CC)
	1368	1362		Wagging CH_2, rocking CH,OH plane bending of aromatic CCH, Rocking (NH), ring deformation[(a)]
1447	1433		1436, 1461	bending CH_3, OH and C=C stretching, rocking (NH)(CH), ring deformation[(a)]
1522, 1547	1543	1531, 1555	1537	C=C stretching, rocking (NH)(CH), scissoring (NH2), rocking (NH)[(a)]
1617	1615	1617	1617	C=C stretching, Scissoring (NH2), rocking (NH) [(a)]

(a) Castillo, J.J.; Rozo, C.E.; Bertel, L.; Rindzevicius, T.; Mendez-Sanchez, S.C.; Martínez Ortegac, F.; Boisen, A. *J. Braz. Chem. Soc.*, **2016**, *27*, 971-977.
